# OfWRKY33 binds to the promoter of key linalool synthase gene *OfTPS7* to stimulate linalool synthesis in *Osmanthus fragrans* flowers

**DOI:** 10.1093/hr/uhaf155

**Published:** 2025-06-16

**Authors:** Wan Xi, Meng-Yu Jiang, Lin-lin Zhu, Xu-Mei Zeng, Huan Ju, Qin-Lian Yang, Ting-Yu Zhang, Cai-Yun Wang, Ri-Ru Zheng

**Affiliations:** National Key Laboratory for Germplasm Innovation & Utilization of Horticultural Crops, Huazhong Agricultural University, Wuhan 430070, China; College of Horticulture and Forestry Sciences, Huazhong Agricultural University, Wuhan 430070, China; Key Laboratory of Plant Germplasm Enhancement and Specialty Agriculture, Wuhan Botanical Garden, Chinese Academy of Sciences, Wuhan 430074, China; National Key Laboratory for Germplasm Innovation & Utilization of Horticultural Crops, Huazhong Agricultural University, Wuhan 430070, China; College of Horticulture and Forestry Sciences, Huazhong Agricultural University, Wuhan 430070, China; National Key Laboratory for Germplasm Innovation & Utilization of Horticultural Crops, Huazhong Agricultural University, Wuhan 430070, China; College of Horticulture and Forestry Sciences, Huazhong Agricultural University, Wuhan 430070, China; National Key Laboratory for Germplasm Innovation & Utilization of Horticultural Crops, Huazhong Agricultural University, Wuhan 430070, China; College of Horticulture and Forestry Sciences, Huazhong Agricultural University, Wuhan 430070, China; National Key Laboratory for Germplasm Innovation & Utilization of Horticultural Crops, Huazhong Agricultural University, Wuhan 430070, China; College of Horticulture and Forestry Sciences, Huazhong Agricultural University, Wuhan 430070, China; National Key Laboratory for Germplasm Innovation & Utilization of Horticultural Crops, Huazhong Agricultural University, Wuhan 430070, China; College of Horticulture and Forestry Sciences, Huazhong Agricultural University, Wuhan 430070, China; National Key Laboratory for Germplasm Innovation & Utilization of Horticultural Crops, Huazhong Agricultural University, Wuhan 430070, China; College of Horticulture and Forestry Sciences, Huazhong Agricultural University, Wuhan 430070, China; National Key Laboratory for Germplasm Innovation & Utilization of Horticultural Crops, Huazhong Agricultural University, Wuhan 430070, China; College of Horticulture and Forestry Sciences, Huazhong Agricultural University, Wuhan 430070, China; National Key Laboratory for Germplasm Innovation & Utilization of Horticultural Crops, Huazhong Agricultural University, Wuhan 430070, China; College of Horticulture and Forestry Sciences, Huazhong Agricultural University, Wuhan 430070, China

## Abstract

Volatile aroma compounds make significant contributions to human perception of flowers. *Osmanthus fragrans* is a famous aroma plant, and linalool is proved to be the dominant aroma active compound. Although some terpene synthases have been characterized, a comprehensive study of the hub metabolic gene and its transcriptional regulation remain to be revealed. Here, we selected a specific cultivar Boyeyingui with the highest content of linalool among 20-wide-cultivated cultivars for genome and transcriptome sequencings. Among the 25 new putative *OfTPSs,* only *OfTPS6*, *OfTPS7* could exclusively produce linalool *in planta*. Biochemical analysis demonstrated that OfTPS6, OfTPS7 were able to catalyze geranyl diphosphate into linalool and a small proportion of other monoterpenes *in vitro*. Spatial and temporal correlation analysis further confirmed the expression level of *OfTPS7* was strongly correlated with linalool content in a panel of 20 cultivars, suggesting *OfTPS7* was the essential linalool synthase gene. Combined with yeast one-hybrid screen and weighted correlation network analysis, a nucleus-localized transcriptional factor OfWRKY33 was identified as a prospective modulator. Y1H, LUC, and EMSA demonstrated that OfWRKY33 directly bound to the W-box of *OfTPS7* promoter to stimulate its transcription. OfWRKY33 could coordinately induce the expressions of *OfTPS7* and 1-deoxy-d-xylulose 1, thereby promoting the linalool formation*.* The results first identified the key linalool synthase gene *OfTPS7* and a novel transcription factor playing a role in the complex regulatory network of linalool biosynthesis in *O. fragrans* flowers.

## Introduction

Aroma compounds are crucial for development and interaction with environment for plants [1]. They also make great contribution to consumers’ perception of flowers. Aroma compounds derived from the plants are widely extracted and applied in a broad range of industries [2–4]. However, due to the complex biochemical biosynthesis process and regulation mechanism, improving the aroma traits is always a considerable challenge for horticultural plants.

*Osmanthus fragrans* is a famous aroma plant, which has a history in China dating back over 2500 years [5]. In most cultivars, approximately 70% of total aroma compounds are terpenoids synthesized through the plastid-localized 2-C-methy-D-erythritol 4-phosphate (MEP) pathway [6–10]. Linalool is an important aroma active compound in flowers of diverse *O. fragrans* cultivars, imparting characteristic floral scents to fresh flowers and its essential oil [4, 11, 12]. Terpene synthases (TPSs) are the vital enzymes directly converting GPP into linalool and other monoterpenes [13–16]. Previous studies have identified candidate linalool synthase genes *OfTPS1/2/5* through homology alignment with *Arabidopsis thaliana* orthologs and functionally characterized by *in vivo* and *in vitro* experiments [17–20]. However, genome-wide analysis coupled with population resequencing data revealed significant allelic diversity within the *OfTPS* gene family across *O. fragrans* cultivars [17–21]. This interspecific variation underscores the necessity for systematic functional characterization and spatiotemporal expression profiling of the entire *OfTPS* gene repertoire. Such comprehensive analyses would not only clarify the molecular mechanisms underlying linalool biosynthesis but also pinpoint the dominant *OfTPSs* responsible for monoterpene production across most *O. fragrans* cultivars, thereby advancing targeted metabolic engineering strategies in aromatic plant breeding. Although plants possess a considerable number of *TPSs* in the genome, a limited proportion of *TPSs* are capable of producing terpenoids [22, 23]. For instance, in *Freesia × hybrida*, *FhTPS1* is responsible for linalool formation, while *FhTPS4*, *FhTPS6,* and *FhTPS7* are bifunctional genes producing mono-/sesqui-terpenes simultaneously [13]. To gain a comprehensive understanding of terpenoid biosynthesis in plants, it is essential to conduct genome sequencing along with *in vivo* and *in vitro* functional identification [23, 24].

Recent studies have revealed that transcription factors such as MYB, AP2/ERF, bHLH, MADS, and WRKY play a regulatory role in terpenoid biosynthesis by directly interacting with the promoters of key pathway genes [25–30]. FhMYB21L2-mediated linalool synthesis by interacting with the MYBCORE site on the promoter of *FhTPS1* [25]. OfMYB21 could bind to the promoter of *OfTPS2* and positively affected linalool synthesis [20]. OfMYB1R114 enhanced the production of the β-ionone aroma compound through interaction with the *OfCCD4* promoter and upregulating its expression in *O. fragrans* [31]. WRKY transcription factors play a widespread role in plant development and growth regulation by binding to the W-box cis-acting element [29, 30]. Recent studies have shown that WRKY are also participating in the synthesis and control of secondary metabolites. For instance, *CrWRKY42* enhances carotenoid accumulation by increasing the expression of multiple carotenoid biosynthetic genes [30]. About, 154 *OfWRKY* genes were screened with WRKY domains in *O. fragrans* genome and 8 *OfWRKY* genes presented flower-specific expression patterns [32]. However, their potential function and transcriptional regulation of aroma compounds in *O. fragrans* flowers remain elusive.

This study aimed to pinpoint the principal linalool synthase gene and unravel its regulatory network. To achieve this, we first selected the cultivar ‘BBYG’—exhibiting the highest linalool levels among 20 tested cultivars—for genomic and transcriptomic profiling. Through functional characterization and expression profiling of all *OfTPS* family members, *OfTPS7* emerged as the pivotal gene governing linalool production. Departing from conventional homology-based single-gene analyses, our genome-wide evaluation of *OfTPS* homologs not only resolved longstanding uncertainties in functional annotation but also demonstrated OfTPS7’s conserved role in linalool biosynthesis across diverse cultivars. Next, employing *OfTPS7* as a yeast one-hybrid (Y1H) screening probe and combining it with WGCNA of transcriptomic data, we identified OfWRKY33 as a central transcriptional activator. This regulator directly targets the W-box motif in the *OfTPS7* promoter, activating its expression while coordinately modulating multiple MEP pathway genes to enhance linalool synthesis. Strikingly, OfWRKY33’s expression profile across all 20 cultivars correlated tightly with linalool accumulation, reinforcing its evolutionary conservation in fragrance biosynthesis. Moving beyond earlier fragmented studies limited to individual genes or single cultivars, our findings establish an integrated TPS-WRKY-MEP regulatory axis. This framework not only deciphers the molecular basis of linalool production but also equips breeders with precise genetic targets for engineering superior aromatic traits in *O. fragrans*.

## Results

### Linalool is the key characteristic aroma compounds of *O. fragrans* flowers

**Figure 1:**
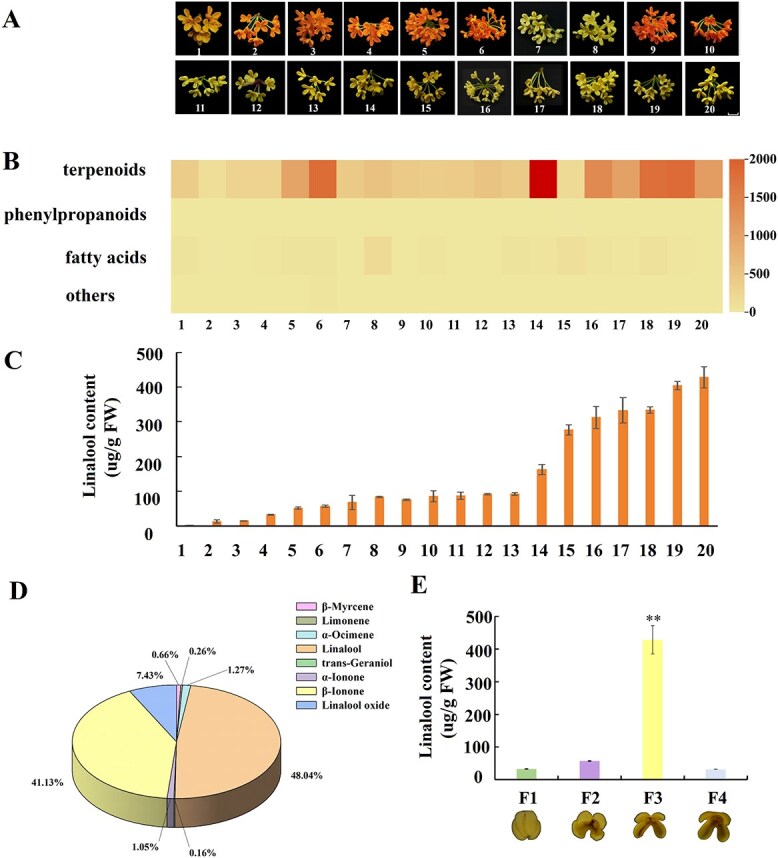
Volatile aroma compounds in *O. fragrans* flowers. **A** Full blossoming flowers of 20 wide-cultivated *O. fragrans* cultivars; 1: Chenghongdangui, 2: Zhuangyuanhong, 3: Xiangshandangui, 4: Zhushagui, 5: Hongyanningxiang, 6: Jiaorong, 7: Liuyeyingui, 8: Huangchuanjingui, 9: Lianzidangui, 10: Fujianhong, 11: Jinqiuzao, 12: Guizhousijigui, 13: Ruanyejingui, 14: Ziyue, 15: Suiyin, 16: Fudingzhu, 17: Dayehuang, 18: Danzhuang, 19: Mijieyingui, 20: Boyeyingui. **B** Heatmap of different volatile aroma compound contents in 20 cultivars; Scale bar = 1 cm. **C** Linalool contents of the full blossoming stage flowers in 20 cultivars. **D** Proportion of different terpenoids in full blossoming flowers of BBYG. **E** Linalool contents of the four blossoming stages of BBYG, F1: bud stage; F2: initial blossoming stage; F3: full blossoming stage; F4: late blossoming stage. All experiments were performed three times, each containing three biological replicates. The error line represents the average ± SD, and the asterisk represents the significant difference analysis compared with the control group, evaluated by one-way ANOVA. ^*^P < 0.05, ^**^P < 0.01, ^***^P < 0.001

The volatile aroma compounds in fully bloomed flowers of 20 widely cultivated *O. fragrans* cultivars were detected using HS-SPME combined with GC–MS (Fig. 1A). Terpenoids, phenylpropanoids, fatty acid derivatives, and other aroma compounds were found. Terpenoids were the predominant aroma compounds across all cultivars (Fig. 1B). Linalool and its oxides were the dominant aroma active compounds due to its high contents and odor activity values (OAVs, Supplementary Table S1, Supplementary Fig. S1). They could impart noticeable floral fragrance to *O. fragrans* flowers. The specific cultivar ‘BYYG’ was subjected for further study of linalool biosynthesis due to its maximum linalool content among 20 cultivars (Fig. 1C). Linalool and its oxides accounted for 48% and 7.4% of the terpenoids in ‘BYYG’, respectively (Fig. 1D). In addition, temporal analysis demonstrated that linalool and its oxides increased from bud stage and achieved its maximum content in the full blossoming stage (Fig. 1E). Thus, our study selected the specific cultivar ‘BYYG’ for genome and transcriptome profiles in an attempt to elucidate the complex linalool biosynthesis in *O. fragrans* flowers.

### Identification of the TPS gene family

**Figure 2:**
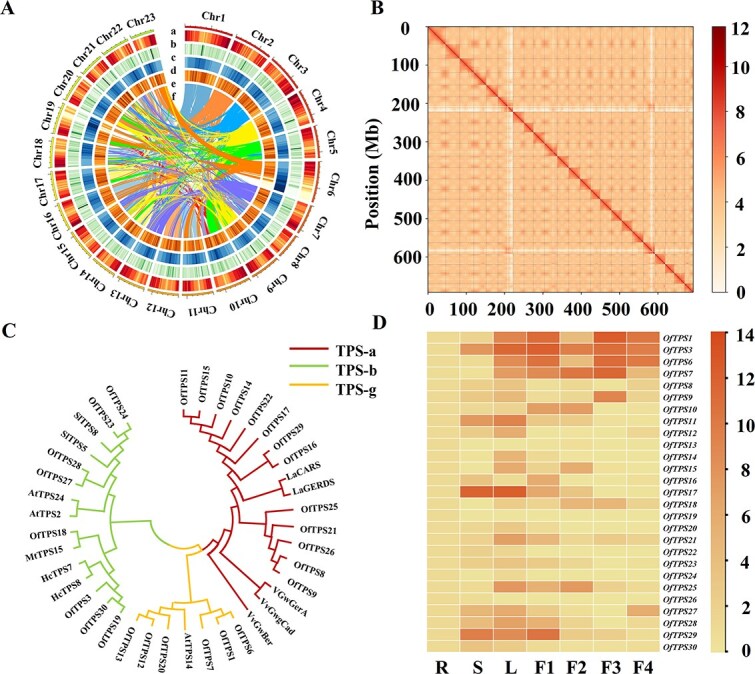
Genomic sequencing and *OfTPSs* sequence analysis of ‘BBYG’. **A** Genomic characteristic map of 'BBYG', a: Chromosome number. b: Gene density; c: LINE; d: LTR; e: DNA transposon; f: Chromosomal collinearity. **B** Hi-C interaction diagram of the genome of ‘BBYG’. **C** Phylogenetic analysis of TPSs from *O. fragrans* and other plants by maximum likelihood method using MEGA7 software. **D** Heatmap of *OfTPSs* expressions at different tissues root, stem, young leaves and different blossoming stages (F1–F4) detected by RT-qPCR

To initiate the investigation of the *OfTPSs* family in *O. fragrans*, ‘BYYG’ flowers with the highest content of linalool were subjected to genome sequencing and transcriptome sequencing of four blossoming stages (Figs 1E and 2AB). Multiple sequencing technologies were employed to assemble a reference genome for *O. fragrans*. The sequencing generated 264.92 Gb of HiFi clean data and 49.86 Gb of SMRT clean data were generated, respectively. K-mer-based statistics revealed the genome size was 711.42 Mb, characterized by a high heterozygosity of 1.23%. The assembled genome reached 726 Mb, featuring a contig N50 of 18.83 Mb (Supplementary Table S2), significantly exceeding those of ‘Liuyeyingui’ (OFL, contig N50 = 2.36 Mb) [33] and ‘Rixianggui’ (OFR, contig N50 = 1.60 Mb) [21]. Using Hi-C sequencing technology, we mapped 98.21% of the sequences to 23 chromosomes (Fig. 2A and B). Owing to the high-quality assembly, BUSCO assessment revealed 98.20% genome completeness. The predicted genes encompassed 97.50% of the evolutionarily conserved core proteins in the eudicot lineage, surpassing the values observed in the other two *O. fragrans* genomes (94.50% and 96.80%, respectively). The elevated mapping rate of ISO-seq reads and the elevated BUSCO score indicated the exceptional completeness and accuracy of the assembled genome. In conclusion, the assessments revealed that the *O. fragrans* ‘BYYG’ genome (OFB) exhibited higher quality in both assembly and annotation (Table 1). Based on the genome, transcriptome sequencing of four developmental stages of flowers was conducted as well. After library construction, Illumina sequencing and assembly, approximately 31.81, 30.8, 33.55, and 33.35 million total clean reads for four samples were generated, respectively (Supplementary Table S3), the above data indicate that the transcriptome data were qualified for subsequent analysis.

**Table 1:** Comparisons of genome assemblies and annotations of *O. fragrans* ‘Boyeyingui’ (OFB), and the published *O. fragrans* ‘Rixianggui’ (OFR), ‘Liuyeyingui’ (OFL), ‘Zhuangyuanhong (ZYH), and ‘Yulianyinsi’ (YLYS)

Content	OFR genome	OFL genome	ZYH genome	YLYS genome	OFB genome
Genome size (Mb)	740.71	733.26	732.21	728.92	726.605
Contig N50 (Mb)	1.6	2.36	21.38	19.15	18.83
Annotated gene number	45 542	41 252	45 236	42 324	45 370
Assembled BUSCOs	96.10%	96.70%	98.50%	98.40%	98.20%
Heterozygosity	1.45%	1.17%	1.02%	1.12%	1.23%
Reference	[21]	[33]	[24]	[24]	This study

Due to lack of genome sequences, only 3 *OfTPSs* were screened by NCBI blast and identified as linalool synthase genes. In this study, 27 *OfTPSs* were annotated, including 25 novel candidate genes identified through HMM scanning and BLASTp searches, which have not yet been functionally characterized. *OfTPS1* and *OfTPS3* were previously clarified and functionally identified as linalool synthase genes [17]. Twenty-five full-length genes encoding putative proteins with 260 to 683 amino acids were amplified and designated as *OfTPS6-30* according to their chromosomal position (Supplementary Table S4). Most *OfTPSs* were identified in clusters on chromosomes I, II, V, VI, and XIX, indicating multiple duplication and neofunctionalization events on these chromosomes. (Supplementary Figs S2 and S3). *OfTPSs* were classified into three subgroups through phylogenetic analysis: TPS-a (13), TPS-b (7), and TPS-g (5) (Fig. 2C). According to amino acid sequence alignment, *OfTPS1/6/7/12/13/20* belonging to TPS-g subfamily were typical lack of RRX_8_W, which was responsible for cyclization reactions (Supplementary Fig. S4). All the other *OfTPSs* contained all the conserved elements including RRX_8_W, DDXXD, and NSE/DTE motifs, which play a fundamental role in binding Mg^2+^ or Mn^2+^ cofactors. Our genomic analysis of ‘BYYG’ and re-sequencing of other cultivars revealed that coding sequences (CDS) of *OfTPSs* were structurally conserved across cultivars (Supplementary Tables S5 and S6). This conservation suggested that structural variations in *OfTPSs* (e.g. mutations, InDels) were unlikely to explain the observed differences in linalool content. Instead, we proposed that differential expression levels of *OfTPSs* and/or functional cooperativity among specific TPS isoforms were key drivers.

To correlate the expression of MEP pathway genes with volatile linalool accumulation during flower development, FPKM values of putative genes were analyzed in four blooming stages based on transcriptome sequencing, with 37 candidate genes implicated in the MEP and mevalonate (MVA) pathways. Expression levels of candidate genes in the MEP pathway were significantly higher than those in the MVA pathway, aligning with the higher concentrations of monoterpenes found in *O. fragrans* flowers. (Supplementary Fig. S5 and Table S7). RT-qPCR was carried out to examine the temporal and spatial patterns of expression of all the *OfTPSs*. Six *OfTPSs* (*OfTPS1*/*3*/*6/7/9/29*) achieved high expressions in flowers compared with other tissues (Fig. 2D). Remarkably, the transcript level of *OfTPS7* increased significantly from the bud stage to full blossoming and then decreased, matching the patterns of volatile linalool emission. (Fig. 2D).

### Overexpression of *OfTPS6* and *OfTPS7* increase production of linalool both *in vitro* and *in vivo*

**Figure 3:**
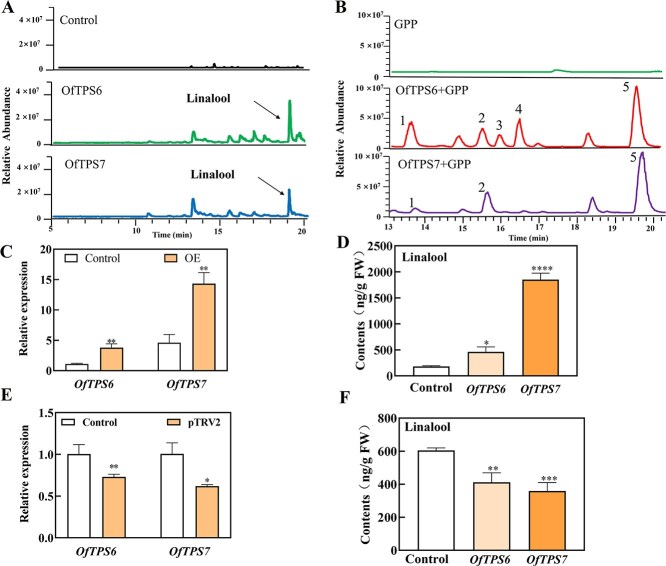
Functional validation of *OfTPS6* and *OfTPS7 in vivo* and *in vitro.*  **A** Aroma compound analysis of tobacco leaves of control, *OfTPS6* and *OfTPS7* overexpressed groups. **B** Extracellular enzyme function of OfTPS6 and OfTPS7 (GPP as substrate), 1: β-Myrcene; 2: D-limonene; 3: α-Pinene; 4: β-Ocimene; 5: Linalool. **C** Expressions of *OfTPS6* and *OfTPS7* in *O. fragrans* by RT-qPCR, Control: flowers were injected with empty vector, OE: flowers were injected with *OfTPS6* and *OfTPS7* overexpression vectors, respectively. **D** Linalool contents of *O. fragrans* flowers in control, overexpressed *OfTPS6*, *OfTPS7* groups. **E**  *OfTPS6* and *OfTPS7* expression levels in *O. fragrans* flowers in control and silencing groups. **F** Linalool contents of *O. fragrans* flowers in control, silenced *OfTPS6*, *OfTPS7* groups. All experiments were performed three times, each containing three biological replicates. The error line represents the average ± SD, and the asterisk represents the significant difference analysis compared with the control group, evaluated by one-way ANOVA. ^*^P < 0.05, ^**^P < 0.01, ^***^P < 0.001

To validate the functions of *OfTPSs*, all the 25 new *OfTPSs* (*OfTPS6*-*OfTPS30*) were cloned and transiently transformed into tobacco leaves and *O. fragrans* flowers to determine whether they were capable of producing linalool. In the tobacco leaves, only *OfTPS6* and *OfTPS7* could synthesize linalool (Fig. 3A), whereas other genes did not yield any products (Supplementary Fig. S6). To verify the enzymatic functions of OfTPS6 and OfTPS7, substrate specificity analyses were performed with GPP and farnesyl diphosphate (FPP) as substrates, respectively. The recombinant proteins of OfTPS6 and OfTPS7 were expressed in *Escherichia coil* and purified as soluble proteins. *In vitro* enzymatic assays showed that OfTPS6 and OfTPS7 multifunctional enzymes producing multiple products. Notably, OfTPS6 mainly converted GPP into linalool and a few by-products such as β-myrcene, D-limonene, α-pinene, and cis-β-ocimene (Fig. 3B). OfTPS7 could catalyze GPP into linalool along with a small amount of β-myrcene and D-limonene (Fig. 3B). OfTPS6 and OfTPS7 could not produce any volatile aroma compounds in the incubation with FPP as precursor (Supplementary Fig. S7). In addition, *OfTPS7* was proved to located in plastids where monoterpenes were produced, whereas *OfTPS6* was found to be localized in the cytosol (Supplementary Fig. S8). These results clearly showed the linalool biosynthesis function of *OfTPS6* and *OfTPS7* both *in vitro* and *in vivo*.

Overexpressing *OfTPS6* transiently in flowers resulted in approximately 3.43-fold increase in its transcript level and 5.35-fold increase of volatile linalool and its oxides relative to empty vector control (Fig. 3C and D, Supplementary Fig. S9). Moreover, transient overexpression of *OfTPS7* in flowers resulted in an approximately 3.10-fold increase in its transcript level and a 7.7-fold rise in volatile linalool and its oxides compared to the empty vector control. (Fig. 3C and D, Supplementary Fig. S9). On the contrary, linalool and its oxides were detected at significantly lower concentrations in the *OfTPS6* and *OfTPS7* transient silencing group relative to the control group (Fig. 3E and F).

### *OfTPS7* plays a crucial role in linalool biosynthesis in diverse cultivars

**Figure 4:**
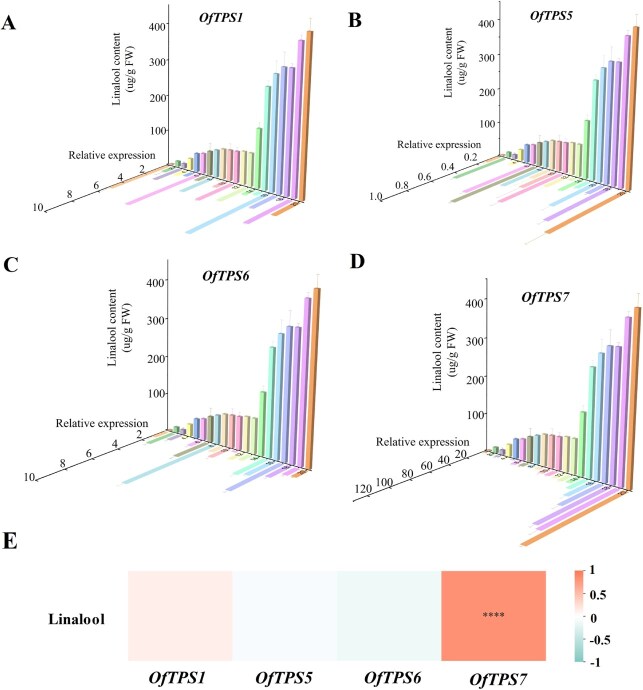
Correlation analysis of linalool synthase gene expressions with linalool and its oxides contents in 20 cultivars. **A-D** Swallow-tail plot analysis of the *OfTPS1, OfTPS5, OfTPS6, OfTPS7* expression levels and linalool contents in different cultivars. **E** Correlation analysis between the expression level of high expression linalool synthase gene in flowers and the content of linalool. The error line represents the average ± SD, and the asterisk represents the significant difference analysis compared with the control group, evaluated by one-way ANOVA.

Linalool is synthesized through the MEP pathway, with TPS enzymes playing a critical role, the variation in linalool content among cultivars is indeed a complex phenomenon, likely influenced by multiple factors, including genetic and environmental factors. Our re-sequencing analysis revealed that the coding sequences (CDS) of *OfTPSs* genes are relatively conserved across the 20 cultivars, and importantly, all the protein sequences contain the conserved TPS domains, such as the DDXXD and NSE/DTE motifs, which are crucial for TPS activity (Supplementary Tables S7 and S8). We detected the transcription levels of all the linalool synthase genes *OfTPS1/2/5/6/7* and linalool contents in 20 cultivars to explore the key linalool synthase genes in *O. fragrans*. The results showed that *OfTPS2* exclusively expressed in two cultivars ‘BBYG’ and ‘MJYG’. *OfTPS1*, *OfTPS5,* and *OfTPS6* expressed in most cultivars, but their correlation coefficients with linalool content were less than 0.5. Only *OfTPS7* expressed in all 20 cultivars and its co-expression coefficient with linalool content reached the maximum value at 0.82 (Fig. 4A–D, Supplementary Tables S7 and S8). In addition, among the associations between the expression levels of *OfTPS1*, *OfTPS2*, and *OfTPS7* and linalool content in 'Boyeyingui', TPS7 showed the highest correlation coefficient, reaching 0.94 (Supplementary Table S9). Additionally, spatial analysis demonstrated that *OfTPS7* achieved high expression levels at the initial and full blossoming stages in flowers, in parallel with the linalool release (Fig. 4D and E). Therefore, we concluded that *OfTPS7* played a fundamental role in linalool biosynthesis in *O. fragrans* flowers.

### Y1H assay combined with WGCNA facilitate the screening of the potential transcription factor *OfWRKY33*

**Figure 5:**
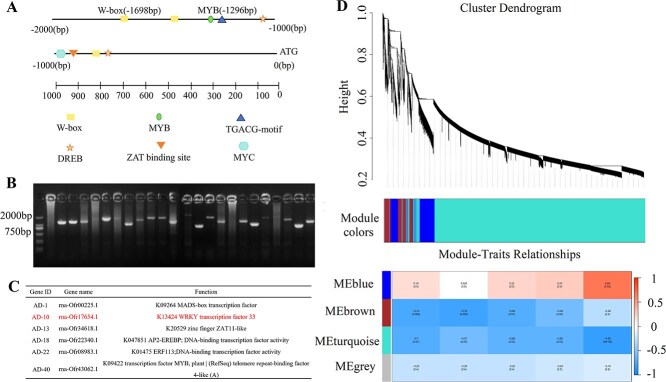
Screen of potential regulators of *OfTPS7* combined by Y1H and WGCNA. **A** Analysis of cis-acting elements in *OfTPS7* promoter. **B** Detection of positive bacteria in yeast single impurity screening library. **C** Functional annotation of yeast single impurity screening library positive bacteria. **D** WGCNA analysis of transcriptome at different flowering stages of ‘BBYG’

**Figure 6:**
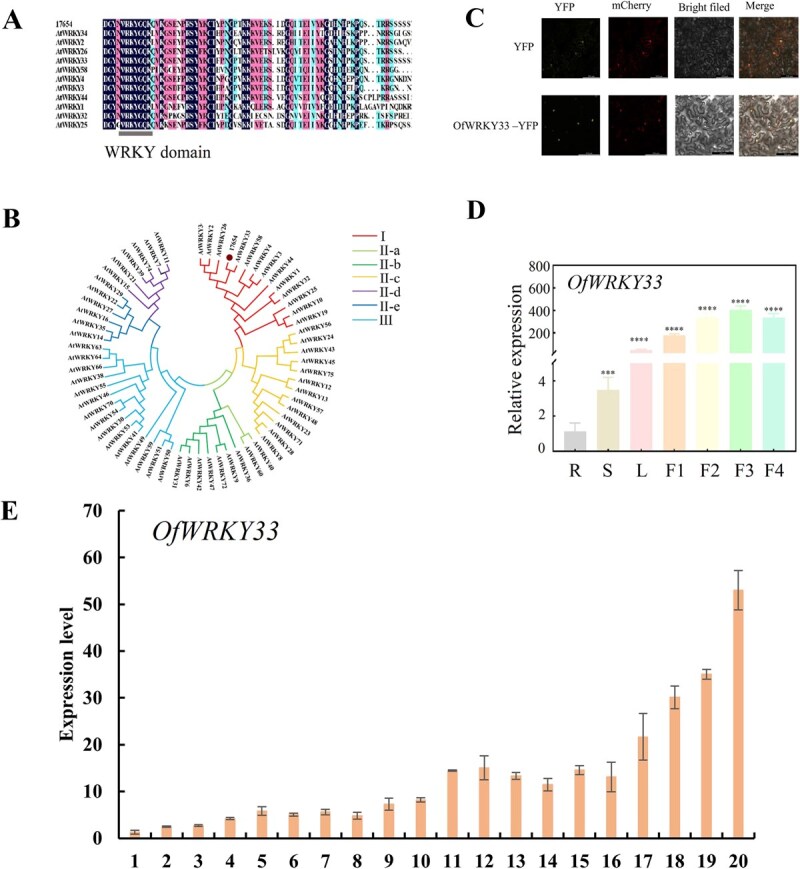
Expression profiles, sequence characterization and subcellular localization of *OfWRKY33*. **A** Partial sequence alignment between OfWRKY33 and AtWRKYs showing the conserved domain **B**. Evolutionary relationships among WRKY proteins from *O. fragrans* and other species were analyzed. The phylogenetic tree was generated using the maximum likelihood approach in MEGAX software. TPS protein information are available in Supplementary Table S5. **C**  *OfWRKY33* subcellular localization, YFP fluorescence indicated the location of each fusion protein , the location of nucleus was determined by nuclear localization marker . **D** Expression patterns of *OfWRKY33* in different tissue parts and flowering stages of 'BBYG'. **E** Expression levels of *OfWRKY33* in the 20 cultivars. The 'Merged' column displayed the combined fluorescence signals from all channels. Bars = 100 μm. All experiments were performed three times, each containing three biological replicates. The error line represents the average ± SD, and the asterisk represents the significant difference analysis compared with the control group, evaluated by one-way ANOVA ^*^P < 0.05, ^**^P < 0.01, ^***^P < 0.001

After pinpointing the key gene that codes for the enzyme involved in linalool production, our subsequent focus shifted to understanding the regulatory mechanisms of *OfTPS7*. To identify potential regulators, a Y1H screening was conducted with the *OfTPS7* promoter serving as the bait, targeting a cDNA library constructed from *O. fragrans* floral tissues. Six potential candidates, including members of the WRKY, ERF, MADS, ZAT, and MYB families, were identified, with one specifically classified as a transcription factor from the WRKY family (Fig. 5A–C, Supplementary Table S10). To explore the key genes associated with linalool biosynthesis, transcriptome sequencing was performed on *O. fragrans* flowers at four critical developmental stages, aiming to identify potential regulatory factors. DEGs were determined using log_2_ FC of ±1 and an adjusted *P*-value <0.01. A total of 19 199 non-redundant DEGs were retained for WGCNA after filtering, leading to four enriched modules related to linalool and other terpenoids, respectively. The correlation analysis between modules and traits indicated that the blue module, comprising 1890 genes, showed a strong link to linalool synthesis. (*r* = 0.66, *P* = 0.02) (Fig. 5D). In addition, A stricter threshold (|Log_2_ FoldChange| ≥ 1.5) was applied to the 1890 genes in the blue module, narrowing the pool to 1159 genes with pronounced expression changes (Supplementary Fig. S10A and B). Venn diagram analysis identified 26 high-confidence candidate genes consistently differentially expressed across all developmental stages. Among them, the correlation coefficients of 6 TFs with *OfTPS7* were higher than 0.85 and RT-qPCR results further confirmed that the expression patterns of four TFs were generally parallel with *OfTPS7*(Supplementary Fig. S10C). Spatial and temporal analysis revealed that *OfWRKY33* not only achieved higher transcript level in flowers than in stem, young leaves and root, but also performed similar trend with linalool release in flowers (Fig. 6A). The expression profile of *OfWRKY33* was further analyzed across 20 cultivars, revealing significant positive correlations with both linalool content (*r* = 0.96, *P* < 0.001) and *OfTPS7* expression levels (*r* = 0.90, *P* < 0.001) (Fig. 6E). Meanwhile, Y1H assay also showed potential interaction between *OfWRKY33* and *OfTPS7* (Fig. 5C). The data suggested that *OfWRKY33* might be connected with linalool biosynthesis.

### OfWRKY33 is a nucleus-located transcription factor

*OfWRKY33* (GenBank accession PP598872; *O. fragrans* genome ID Ofr17654) featured a complete cDNA sequence of 1468 bp, which encoding a 489 amino acid protein with a conserved WRKYGQK domain located near its C-terminal region (Fig. 6B). There was a 50% amino acid sequence similarity between OfWRKY33 and *A. thaliana* AtWRKY33 (GenBank NP_181381.2) (Fig. 6C). The predicted molecular weight of WRKY33 was 53.796 kDa. According to phylogenetic analysis, OfWRKY33 was grouped into the WRKY-I subfamily (Supplementary Table S11). To investigate the subcellular localization, a *CaMV35S*:*OfWRKY33*-*YFP* construct and a *CaMV35S*: *YFP* control construct were introduced into *Nicotiana benthamiana* leaves, respectively. Fluorescence microscopic analysis revealed that *OfWRKY33-YFP* was specifically localized in the nucleus, colocalizing with mCherry, while *CaMV35S: YFP* was uniformly distributed across the entire cell (Fig. 6D).

### *OfWRKY33* enhances the linalool content and affects the expression of some MEP pathway genes in transgenic flowers

**Figure 7:**
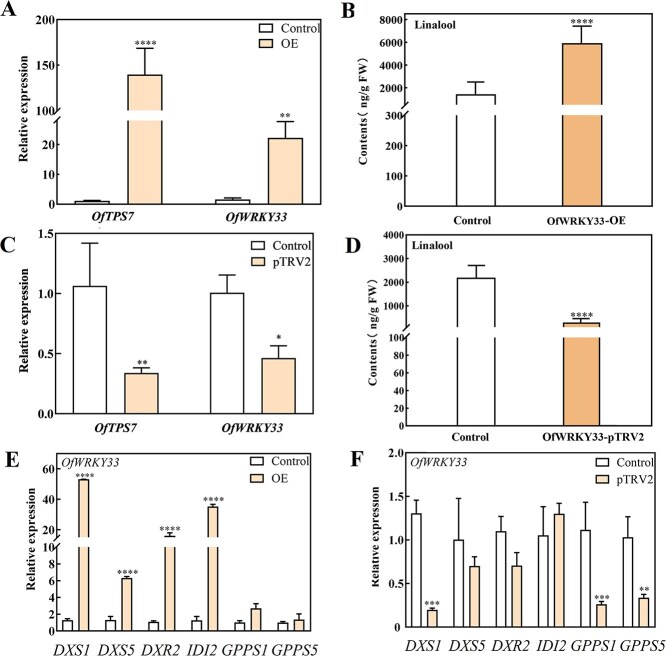
Functional verification of *OfWRKY33* in *O. fragrans* flowers. **A** Gene expression level of *OfWRKY33* and *OfTPS7* in overexpressing strain. **B** Linalool content in overexpressed strains of *OfWRKY33.*  **C** Gene expression level of *OfWRKY33* and *OfTPS7* in silenced strain. **D**  *OfWRKY33* silent line linalool content. **E** Genes expression in MEP pathway in overexpressed and control strains of *OfWRKY33.*  **F** Genes expression in MEP pathway in silenced and control strains of *OfWRKY33*. All experiments were performed three times, each containing three biological replicates. The error line represents the average ± SD, and the asterisk represents the significant difference analysis compared with the control group, evaluated by one-way ANOVA. ^*^P < 0.05, ^**^P < 0.01, ^***^P < 0.001

To further study the roles of *OfWRKY33* in MEP pathway, we applied transiently transgenic experiments in *O. fragrans* flowers. *Agrobacterium tumefaciens* GV3101 containing the plasmid *PK7WG2D-OfWRKY33* and the viral-induced gene silencing (VIGS) plasmid *pTRV2*-*OfWRKY33* vectors were vacuum infiltrated into *O. fragrans* flowers at the initial blossoming stage, respectively. The expressions of *OfWRKY33* and *OfTPS7* were significantly elevated in the overexpressed flowers, resulting in remarkable promotion of linalool and its oxides contents (Fig. 7A and B, Supplementary Fig. S11). The transcript levels of MEP pathway genes were also monitored. Notably, the expressions of *OfDXS1*, *OfDXR2*, and *OfIDI2*, which had higher FPKM values in the transcriptome of different flowering stages of 'BBYG', were also stimulated in *OfTPS7* over-expressed flowers (Fig. 7B and E, Supplementary Fig. S5). The expression levels of other linalool-related genes namely *OfDXS5*, *OfGPPS1,* and *OfGPPS5*, failed to exhibit similar patterns in the *PK7WG2D*-*OfWRKY33* flowers. *OfWRKY33*-silenced flowers contained both remarkably lower gene expressions (especially *OfDXS1*, *OfGPPS1*, and *OfGPPS5*) and linalool contents relative to the control group (7.41 times and 5.85 times) (Fig. 7C and D and Supplementary Fig. S11). These results demonstrated that *OfWRKY33* acts as a positive regulator of linalool biosynthesis.

### *OfWRKY33* directly interacts with the promoters of *OfTPS7* and induces its transcription

**Figure 8:**
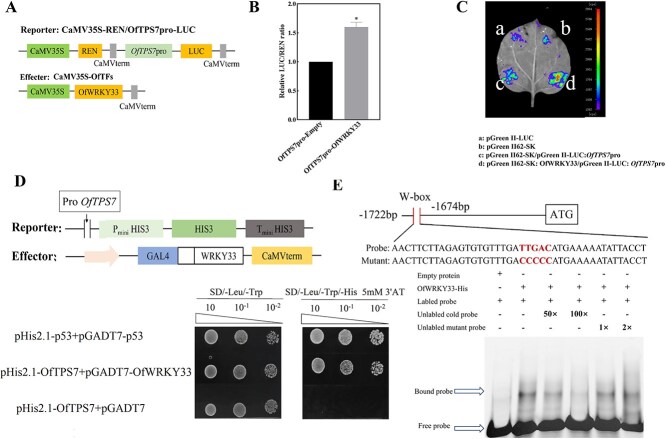
*OfWRKY33* binds directly to the W-box in the *OfTPS7* promoter sequence. **A** Schematic diagram of dual luciferase carrier, the effector vector *OfWRKY33* was constructed on the pGreen-62-SK vector driven by CaMV35S, and the reporter vector *OfTPS7* promoter was constructed on the pGreen II-0800 LUC vector to drive the expression of LUC. **B** The transient fluorescence activity of tobacco indicates that *OfWRKY33* has transcriptional activation activity and can promote the expression of *OfTPS7.*  **C** Relative fluorescence activity of *OfWRKY33* transcriptional activity. **D** Yeast single heterozygous display of interaction between *OfWRKY33* and *OfTPS7* promoter. **E** OfWRKY33 binds directly to the *OfTPS7* promoter, EMSA probe, Mut represents mutation probe, and mutation sites are represented by black dashed boxes, with " + " and "-" indicating the addition or absence of probes or proteins; 50× represents 50-fold unlabled cold probe, 100 × represents 100-fold unlabled cold probe, 1 × represents 1-fold unlabled mutant probe, 2 × represents 2-fold unlabled mutant probe, arrow indicating protein DNA complex or free probe position. All experiments were performed three times, each containing three biological replicates. The error line represents the average ± SD, and the asterisk represents the significant difference analysis compared with the control group, as determined by the Student’s *t*-test. ^*^P < 0.05

To assess the ability of *OfWRKY33* to activate *OfTPS7* expression, we conducted a LUC assay. The *OfTPS7* promoter was ligated into the LUC reporter vector, while the coding sequence of *OfWRKY33* was inserted into the effector vector (Fig. 8A). Relative to the empty vector, *OfWRKY33* markedly enhanced the LUC/LEN ratio under the control of the *OfTPS7* promoter. (Fig. 8B). In addition, LUC imaging assays further verified that *OfWRKY33* induces the *OfTPS7* promoter *in vivo*. A strong fluorescent signal was observed in the coexpression region (*OfWRKY33* + *OfTPS7pro*-Luc), While the control region (EV + *OfTPS7pr*o-Luc) displayed a faint fluorescent signal (Fig. 8C). *OfWRKY33* also demonstrated significant interactions with the promoters of *OfTPS7* in Y1H assays (Fig. 8D). Y1H experiments were also conducted to test the relationship between *OfWRKY33* and *OfDXS1*, *OfDXR2,* and *OfIDI2*, but no direct interaction was observed (Supplementary Figs S12 and S13).

To investigate the DNA-binding specificity of the OfWRKY33 protein, electrophoretic mobility shift assays (EMSA) were conducted using the promoter region of *OfTPS7* as the target sequence. The OfWRKY33 protein was successfully induced and purified for this analysis (Supplementary Fig. S14). The EMSA results revealed a specific shifted band when the recombinant protein OfWRKY33-His was mixed with the labeled probe. Notably, this His-tagged protein-DNA complex band diminished progressively with increasing concentrations of unlabeled wild-type competitor probes, demonstrating concentration-dependent competition. In contrast, the addition of unlabeled mutant probes failed to compete for binding, resulting in retention of the shifted band. These observations conclusively validate the specificity of OfWRKY33 for the W-box motif within the *OfTPS7* promoter *in vitro*.

## Discussion

**Figure 9:**
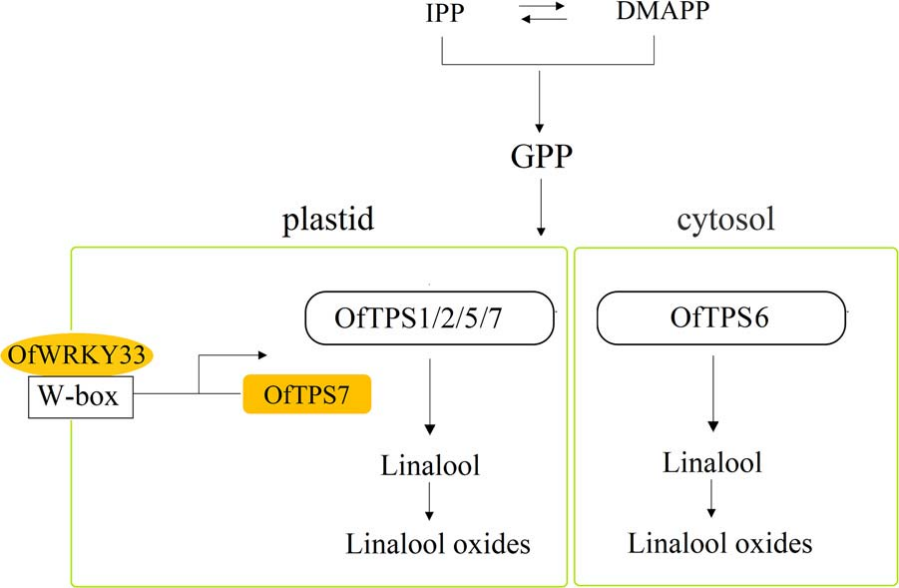
The linalool biosynthesis and its transcriptional regulation in *O. fragrans* flowers. GPP serve as the donor, *OfTPS1/2/5/7* are located in plastid and express in 20 cultivars, whereas *OfTPS6* is located in cytosol and express in 2 cultivars. Among them, *OfTPS7* is a key linalool synthase gene and OfWRKY33 can directly interact with the W-box in the *OfTPS7* promoter to manipulate linalool formation

Terpenoids are abundant aroma compounds in different flowers and linalool along with its oxides are determined as vital aroma active compounds in various plants [34–36]. Despite significant advances in elucidating the biosynthetic pathways of linalool in various species, the hub linalool synthase genes together with their underlying transcriptional regulation are not well understood. Here, we elucidated the dominant role of *OfTPS7* in the synthesis of linalool in multiple cultivars and deciphered the novel OfWRKY33-driven regulatory network of linalool biosynthesis in *O. fragrans* flowers. We demonstrated that OfWRKY33 positively regulates linalool formation by directly interacting with the *OfTPS7* promoter and enhancing the expression of *OfTPS7* and other key pathway genes (Fig. 9).

### Comprehensive study of *OfTPSs* provides global view of terpenoid formation

TPSs serve as key regulators in the biosynthesis of diverse terpenoids in plants [37]. TPS gene family is a mid-size family of highly diversified sequences and functions, comprising roughly 20 to 180 genes in plant genomes [38–41]. The first linalool synthase gene (LIS) from the flowers of *Clarkia breweri* was isolated in 1996 [42]. In recent years, molecular mechanism study of terpenoids is developing rapidly, the characterization of TPSs have been achieved in various plants such as *Rosa hybrida* [14], *Fressia × hybrida* [13], *Lathyrus odoratus* [3], *Dendrobium officinale* [34], *Chrysanthemum indicum* [43], and so on. In fact, only a small proportion of TPSs in genome are responsible for yielding terpenoids in plants. For instance, 2 members out of 40 *AtTPSs* were capable of forming over 20 sesquiterpenes in *A. thaliana* [44]*.* To date, all the *OfTPSs* annotated in the genome have not been thoroughly characterized to gain a comprehensive view of terpenoid formation in *O. fragrans*. Five linalool synthase genes *OfTPS1/2/5/6/7,* belonging to TPS-g subfamily, were identified as linalool synthase genes [17]. *OfTPS3/4* were clustered into TPS-b subfamily. *OfTPS3* was capable of producing trans-β-ocimene, while *OfTPS4* could produce the sesquiterpene α-farnesene.

The composition of terpenoids was closely related to subcellular localization, gene function and gene expression [23, 45]. *OfTPS7* exclusively distributed in plastid, where GPP was dominantly produced as substrate for monoterpenes. However, *OfTPS6* was found to be localized in the cytosol (Supplementary Fig. S8), where a proportion of GPP might originate via crosstalk from MEP pathway to MVA pathway [46]. In addition, *OfTPS7* presented relatively high expression and was closely related with the linalool content in 20 cultivars (Fig. 4). Although *OfTPS2* was identified as linalool synthesis gene, its expression was not detected in 18 cultivars. The correlation coefficient of *OfTPS1/5/6* expression with linalool contents were lower than 0.5. Thus, we proposed that although *OfTPS1/2/5/6/7* produced the overlapping linalool, *OfTPS7* was the core linalool synthase gene in most *O. fragrans* flowers. In specific cultivars, such as *OfTPS2* and *OfTPS5* act cooperatively with *OfTPS7* to modulate linalool production [20]. This functional interplay may arise from cultivar-specific transcriptional or post-transcriptional mechanisms, including promoter cis-element polymorphisms, epigenetic regulation, or interactions with upstream regulators (e.g. transcription factors, hormonal signaling molecules). Additionally, metabolites from the broader MEP pathway such as carotenoids (e.g. α-carotene, β-carotene) and their cleavage products (e.g. α-ionone, β-ionone) may indirectly impact linalool biosynthesis by competing for shared geranyl diphosphate (GPP) precursors, thereby modulating precursor availability for TPSs.

Although OfTPS6/7 could give rise to multiple monoterpenes with incubation of GPP, they could exclusively produce linalool in planta. In this case, the loss of catalytic abilities of OfTPS6/7 might be a consequence of the important biological roles of linalool and its derivatives for *O. fragrans.* Moreover, a remarkable proportion of linalool tended to be converted into glycosylated 8-hydroxylinalool during the late blossoming stage possibly acting as antimicrobial and antiherbivorous defense for the forthcoming fruit [47, 48].

### Characterization of *OfTPSs* lays foundation for future metabolic engineering

Terpenoids such as linalool and its oxides, α-ionone, β-ionone are extremely abundant in *O. fragrans* flowers. They have been widely applied as ingredients in food, cosmetics, therapeutic, and flavoring products [49, 50]. Conventionally, these aroma compounds are obtained from plant extracts using organic solvents or distillation. But the great challenges for industrial utilization of *O. fragrans* flowers are the low flower production, short blossoming period, postharvest treatment, and essential oil extraction technology. It is extremely costly and laborious if high-quality aroma products are required. Thus, microbial biosynthesis has emerged as alternative source with great promise for meeting the increasing demand for terpenoid biomanufacturing [51]. The characterization of functional *OfTPSs* provides useful gene resources for metabolic engineering in *Saccharomyces cerevisiae* and *Yarrowia lipolytica* for the future.

### Transcriptional regulation expands understanding of molecular mechanism of linalool formation

As the terminal biosynthesis gene in terpenoid biosynthesis, *TPS* was the hot topic of aroma compound formation [52]. However, only a limited number of TFs have been identified as directly regulating TPSs. For instance, LaMYC7 directly interacts with the promoter of *LaTPS76* to regulate the biosynthesis of linalool and caryophyllene. in *Lavendula angustifolia* [48]. *SlMYB75* was capable of binding to *SlTPS12*, *SlTPS31*, and *SlTPS35* and suppressing their transcription, thereby negatively affecting the biosynthesis of β-caryophellene and α-humulene in *Solanum lycopersicum* [26]. In flowers of *Freesia hybrida*, FhMYB21L2 enhanced transcription of linalool synthase gene *FhTPS1* through binding to the MYBCORE sites in the promoter [25]. The characterization of upstream transcription factors (TFs) influencing terpenoids is significantly behind compared to color-related compounds like anthocyanin and carotenoid.

Since the functions of *OfTPSs* have been thoroughly elucidated and *OfTPS7* was proved to be closely associated with the linalool formation, the transcriptional regulation has become a new focus in *O. fragrans*. In the current study, WGCNA derived from transcriptomes, yeast single impurity screening library, expression analysis and functional identification were integrated to claim that *OfWRKY33* could positively regulate linalool biosynthesis. Multiple strategies were further conducted to unravel that OfWRKY33 bound directly to a specific W-box in the promoter of *OfTPS7.* The above results provided evidence that OfWRKY33 acted as a direct positive regulator in linalool biosynthesis.

OfWRKY33 also influenced multiple steps throughout the MEP pathway. The expression profiles of *OfDXS1*, *OfDXR2*, and *OfIDI2* aligned with those of *OfWRKY33* in the over-expression and silenced *O. fragrans* flowers. Although these were not housekeeping genes, they were constitutively and highly expressed in *O. fragrans* flowers to provide substrate for linalool formation. Similarly, *SlSCL3* simultaneously stimulated the expressions of *SlTPS* along with upstream isoprenoid precursor pathway genes including *SlHMGS*, *SlHMGR,* and *SlDXS*, which determined the flux through this pathway in *S. lycopersicum* [27]. However, OfWRKY33 did not bind to the promoters of *OfDXS1*, *OfDXR2*, and *OfIDI2* (Supplementary Figs S12–S13), implying that transcriptional activation may require the involvement of other proteins. TFs can form a complex to cooperatively mediate terpenoid biosynthesis [25, 26]. The physical interactions between TFs affected the protein–DNA interactions therefore influencing the gene expression. For example, protein complex of ZmMYC2-ZmEREB92 exhibited stronger DNA binding ability to *ZmTPS6* than ZmMYC2 alone [53]. Future studies should investigate the synergistical regulation concerning the interaction of *OfWRKY33* with other TFs, which will expand our understanding of the regulatory role. WRKYs represent one of the largest families of plant-specific transcription factors, they play pivotal roles in plant defense, growth and development, morphogenesis of trichomes and embryos, hormone signaling and secondary metabolite biosynthesis [23, 30]. Our study proposed that *OfWRKY33* directly interacted with functional genes to regulate the aroma compound biosynthesis, providing new insights for research of WRKY.

## Conclusion

In summary, unlike conventional single-gene methods that depend on homology comparisons with known species, our study comprehensively assessed all *OfTPS* homologs within the genome. Through transgenic validation and expression analyses, we determined that *OfTPS7* serves as the key gene responsible for linalool production. Previous research often underestimated the diversity of *OfTPS* genes, but our findings clarify functional uncertainties by establishing *OfTPS7*’s central role in linalool synthesis across cultivars. Furthermore, we outline a regulatory model for *OfTPS7* transcription. Specifically, OfWRKY33 directly binds to the W-box motif in the *OfTPS7* promoter, while also upregulating genes in the MEP pathway. This study advances beyond earlier single-cultivar or single-gene investigations by uncovering a coordinated TPS-WRKY-MEP regulatory network. The discovery of *OfWRKY33* as a master transcriptional regulator offers a strategic foundation for metabolic engineering, facilitating the precise modulation of fragrance biosynthesis in *O. fragrans*.

## Material and methods

### Plant materials

Petals of 20 *O. fragrans* cultivars (1: Chenghongdangui, 2: Zhuangyuanhong, 3: Xiangshandangui, 4: Zhushagui, 5: Hongyanningxiang, 6: Jiaorong, 7: Liuyeyingui, 8: Huangchuanjingui, 9: Lianzidangui, 10: Fujianhong, 11: Jinqiuzao, 12: Guizhousijigui, 13: Ruanyejingui, 14: Ziyue, 15: Suiyin, 16: Fudingzhu, 17: Dayehuang, 18: Danzhuang, 19: Mijieyingui, 20: Boyeyingui.) were obtained from Xianning (28°32′N, 110°36′E), Hubei Province, China. Among them, the flowers of ‘BBYG’ were collected at different blossoming stages, viz. bud stage (F1), initial blossoming stage (F2), full blossoming stage (F3), and late blossoming stage (F4). Following volatile aroma compound collection, the floral samples were immediately frozen in liquid nitrogen and stored at −80°C for subsequent analysis. All the materials mentioned above were collected with three biological replicates. *N. benthamiana* plants were cultivated in a greenhouse with a photoperiod of 16 hours of light and 8 hours of darkness. The temperature was maintained at 24°C, and the humidity was set at 60%. The plants were exposed to a light intensity of 250 μmol m^−2^ s^−1^.

### Genome sequencing of *O. fragrans* ‘BBYG’

15 μg of high-quality genomic DNA was utilized, which was sourced from flowers at four different stages. We cut genomic DNA into fragments of expected size and sequenced them using pacbio sequence II (Pacific Biosciences, Menlo Park, CA, USA). Hi-C libraries were developed based on prior research [54]. The Hi-C libraries were quantified and sequenced on the MGI-seq platform (BGI, China).

### Genome assembly and annotation

The genome assembly of 'BYYG' was conducted utilizing all subread data generated from SMRT sequencing [55]. Subsequently, in order to improve the quality of genome assembly, we used the PacBio Sequel II platform to sequence the SMRTbell library and read the PacBio-HiFi at a depth of 91.3 × [56]. Finally, contigs were located on 23 chromosomes by contig anchoring, sorting, and positioning. In addition, BUSCO was used to evaluate the integrity and accuracy of the 'BYYG' genome [57].

To identify repetitive sequences in the genome, we employed a combination of homology-based and *ab initio* prediction methods. Additionally, protein-coding genes in the BYYG genome were predicted using RNA-Seq-assisted gene prediction approaches. For functional annotation, predicted genes were compared against the NCBI non-redundant (NR), TrEMBL, InterPro, and Swiss-Prot protein databases using BLASTP (NCBI BLAST v2.6.0+), as well as the KEGG database, to infer gene functions.

### Transcriptome sequencing of flowers in four blossoming stages

In order to obtain effective information for gene annotation, the Iso Seq method was performed using the SMRT sequencing platform to generate full-length transcripts. Petals were collected from the same tree at four flowering stages, and RNA was extracted for library preparation. The RNA-Seq library was constructed using the Clontech SMARTer cDNA Synthesis Kit, incorporating three biological replicates, and subsequently sequenced on the PacBio Sequel II platform (Frasergen Bioinformatics Co., Ltd).

### Identification of differentially expressed genes and co-expression network modules

After filtering out genes with undetected or relatively low expression levels (TPM < 10), differentially expressed genes (DEGs) exhibiting a coefficient of variation (CV > 0.5) were selected. Subsequently, a co-expression network module was constructed using the WGCNA software package [58]. A DEG was declared if was observed. Pairwise comparisons were performed across four developmental stages (F1–F4) through sequential contrasts (F1 vs. F2, F2 vs. F3, and F3 vs. F4). DEGs were identified using a threshold of |Log_2_ FoldChange| ≥ 1 and the associated PFDR<0.05. Candidate genes potentially involved in linalool biosynthesis were identified by extracting DEGs from co-expression modules highly associated with linalool levels. These DEGs were then subjected to further filtering using a threshold of |Log_2_ FoldChange| ≥1.5 to ensure significant differential expression. Subsequently, Venn diagrams were constructed to visualize the overlap of DEGs identified during the four developmental phases. Genes consistently differentially expressed across all four stages were selected as high-confidence candidates for further functional analysis.

### Gene isolation, phylogenetic tree construction, and multiple sequence alignment

We used HMMER software to search for *OfTPSs* and TFs in the genome. *OfTPSs* and TFs containing conserved domains were subjected to the online web tool GSDS2.0 for gene structure analysis. Full-length cDNAs of *OfTPSs* and TFs were amplified using gene-specific primers through PCR (Supplementary Table S12) based on annotated results from the genome database. The cDNA used as template was synthesized from RNA derived from four blossoming stages flowers. Phylogenetic trees were built using the maximum likelihood method in MEGA7.0 software [59], with bootstrap analysis (1000 replicates) to assess the reliability of group assignments.

### RNA extraction and RT-qPCR

Total RNA was extracted using a commercial kit (Aidlab Biotechnology, Beijing, China) and reverse transcribed into cDNA for qPCR analysis. Using β-actin as the internal reference gene, RT-qPCR was conducted on the Roche LightCycler 480 system. The relative expression levels were calculated using the 2^-△△Ct^ method. The RT-qPCR primers used in this study are listed in Supplementary Table S12.

### Transient transformation of *N. benthamiana* leaves and *O. fragrans* flowers

For overexpression analysis, the open reading frames (ORFs) of all *OfTPSs* genes from the ‘BYYG’ genome were amplified and cloned into the PK7WG2D vector, generating *PK7WG2D-OfTPSs* constructs. For gene silencing, virus-induced gene silencing (VIGS) was employed by cloning a specific *OfTPSs* fragment into the Tobacco rattle virus (pTRV2) vector, producing *pTRV-OfTPSs* for targeted knockdown. To functionally validate the TPS genes, we performed transient overexpression assays by introducing all *OfTPS* constructs into tobacco leaves, with *OfTPS6* and *OfTPS7* additionally expressed in *O. fragrans* petals for in planta verification. The sequences of all PCR primers are provided in Supplementary Table S12.

The transient transformation of *OfTPSs* into the *N. benthamiana* leaves was performed as previously described [13, 60]. PK7WG2D-OfTPS6, PK7WG2D-OfTPS7, PK7WG2D-GFP, pTRV2-OfTPS6, pTRV2-OfTPS7, and pTRV1 plasmids were constructed and transferred into *Agrobacterium* GV3101, respectively. Agrobacterium cultures at an OD_600_ of 0.6–0.8 were centrifuged, resuspended in infiltration solution [10 mM 2-(n-morphorinic) ethanesulfonic acid, 10 mM MgCl_2_, 200 μM acetosyringone] and incubated statically at room temperature for 2–3 h. *O. fragrans* flowers at the initial blossoming stages were collected for vacuum infiltration at 0.08 Mpa for 5 min. The residual *Agrobacterium* solutions were removed with sterile water, and the flowers were maintained in 5% sterile sucrose solution in the dark for 60 h. After incubation, RT-qPCR and GC–MS assays were carried out to determine the gene expression and aroma compound contents. The functional identification of OfWRKY33 was also conducted using the above methods.

### Heterologous expression and *in vitro* enzyme activity in *E. coli*

TargetP 2.0 (https://services.healthtech.dtu.dk/services/TargetP-2.0/) and WoLF PSORT were applied to predict the signal peptides of OfTPS6 and OfTPS7. OfTPS6 contained a segment of signal peptide. The truncated *OfTPS6* and *OfTPS7* coding region sequences on the Pet21b vectors were constructed for removal of signal peptide. The vectors were then transformed into Rosetta2 (DE3) cells for further induction and cultivation for 14–16 hours [13]. Purification of recombinant proteins was performed with a His TALON gravity column (Clontech) following the manufacturer’s guidelines, and their purity was confirmed by SDS-PAGE. OfTPSs enzyme assays were performed as described previously [23] using GPP and FPP as substrates. After enzyme assay incubation, products were collected by the SPME for 30 min and subsequently determined by GC–MS.

### Subcellular localization

CDS of *OfTPS6* and *OfTPS7*, excluding the stop codon, were used to create C-terminal GFP fusion constructs, which were then transformed into *A. tumefaciens* GV3101 for preparation. *Agrobacterium* cells containing OfWRKY33-YFP and the nuclear marker mCherry were combined and co-infiltrated into *N. benthamiana* leaves. Fluorescence signals were observed using a confocal laser scanning microscope (TCS SP8, Leica, Germany) three days post-infiltration.

### Y1H analysis

The 1000 bp promoter fragment of *OfTPS7* was cloned into the HIS vector as a bait construct, while the prey cDNA library from *O. fragrans* flowers was prepared following the CloneMiner II cDNA Library Construction Kit protocol. The Matchmaker Gold Y1H Library Screening System (Takara, Kyoto, Japan) was employed for Y1H screening.

To investigate the interactions between OfWRKY33 and *OfTPS7*, *OfDXS1*, *OfDXR2*, and *OfIDI2*, the promoters of *OfTPS7*, *OfDXS1*, *OfDXR2*, and *OfIDI2* were inserted into the plasmid pHIS2 to construct the reporter strain. Simultaneously, the coding sequence of *OfWRKY33* was fused with the Gal4 activation domain (AD) to construct the bait vector pGADT7-OfWRKY33. Positive yeast single colonies were screened on tri-deficient (SD/-Trp/-Leu/-His) medium, and then cultured on the same medium supplemented with different concentrations of 3-amino-1,2,4-triazole (3'AT) at 30°C for 3–5 days. The primers utilized in this study are provided in Supplementary Table S12.

### Dual-luciferase transient expression reporter assay

Promoter motifs were analyzed using the PLACE signal SCAN search software (https://www.dna.affrc.go.jp/PLACE/?action=newplace). The pGreen II 62-SK vector was used to clone the coding sequence of *OfWRKY33*, acting as an effector vector. Reporter vectors were created by fusing the promoter fragments of *OfTPS7* into pGreen II 0800-LUC. Measurements were performed following the manufacturer’s protocol, with six biological replicates for each measurement. Supplementary Table S12 provides a list of primers developed for the creation of transient expression vectors.

### Electrophoretic mobility shift assay

The coding sequence of *OfWRKY33* was inserted into the Pet21b vector to generate the recombinant vector. The fusion vector was heterologously expressed in *E. coli* BL21 (DE3), followed by purification to obtain the recombinant protein. The biotin-labeled probe, synthesized by Tsingke Biotech (Beijing, China), was designed with the specific sequence provided in Schedule S12. His-tagged protein served as a control, and unlabeled probes (identical or mutated oligonucleotides) were used as cold competitors. EMSA was performed following a previously described method [61].

### Qualitative and quantitative analysis of volatile terpenoids

The analysis of volatile compounds was conducted following methods established in our previous studies [47]. Briefly, HS-SPME was used to capture volatile compounds from *O. fragrans* flowers and *N. benthamiana* leaves. The compounds were thermally desorbed and analyzed using a GC–MS system (Thermo Fisher Technologies), with identification achieved by comparing mass spectra to the NIST2017 library and standard samples. Quantitative analysis using methyl nonanoate as internal standard. All the volatile terpenoids tests were repeated for three biological replicates.

### Accession number

Accession numbers for the genomic sequences reported in this study are available through GenBank at: *OfTPS6* (PQ035161), *OfTPS7* (PQ035163), and *OfWRKY33* (PP598872).

### Statistical analysis

All experimental results are presented as mean values ± standard deviation derived from a minimum of four biological replicates. Statistical analyses were performed using GraphPad Prism 8.0 software, with significant differences between groups determined by Student's *t*-test, ^*^P < 0.05, ^**^P < 0.01, ^***^P < 0.001.

## Acknowledgments

We thank Prof. Deng Xiuxin (Huazhong Agricultural University, Wuhan, China) for providing informative guide for this study. This work was funded by the National Natural Science Foundation of China (32172621) and Fundamental Research Funds for the Central Universities (2662024YLPY006 and 2662024FW013).

## Author Contributions

R.Z. conceived and coordinated this project. W.X. and R.Z. designed the research. W.X. performed the experiments and analyzed the data with contributions from L.Z., X.Z. M.J. H.J., Q.Y., T.Y., and W.X. wrote the original manuscript, R.Z. and C.W. reviewed and improved the manuscript. All the authors read and approved the final manuscript.

## Data availability statement

Raw sequencing reads of all Osmanthus fragrans accessions reported in this study have been deposited into the public database of the National Center of Biotechnology Information (NCBI) BioProject under the accession number PRJNA1141249. All data in this study are provided in the article and its supplementary materials

## Conflict of interests

The authors declare that they have no competing interests.

## Supplementary information

Supplementary data is available at *Horticulture Research* online.

## Supplementary Material

Web_Material_uhaf155
