# Supplementary figures and images for: OfWRKY33 binds to the promoter of key linalool synthase gene OfTPS7 to stimulate linalool synthesis in Osmanthus fragrans flowers
Source: Hortic Res. 2025 Jun 16;12(9):uhaf155. doi: 10.1093/hr/uhaf155 (PMC12373639; doi:10.1093/hr/uhaf155)

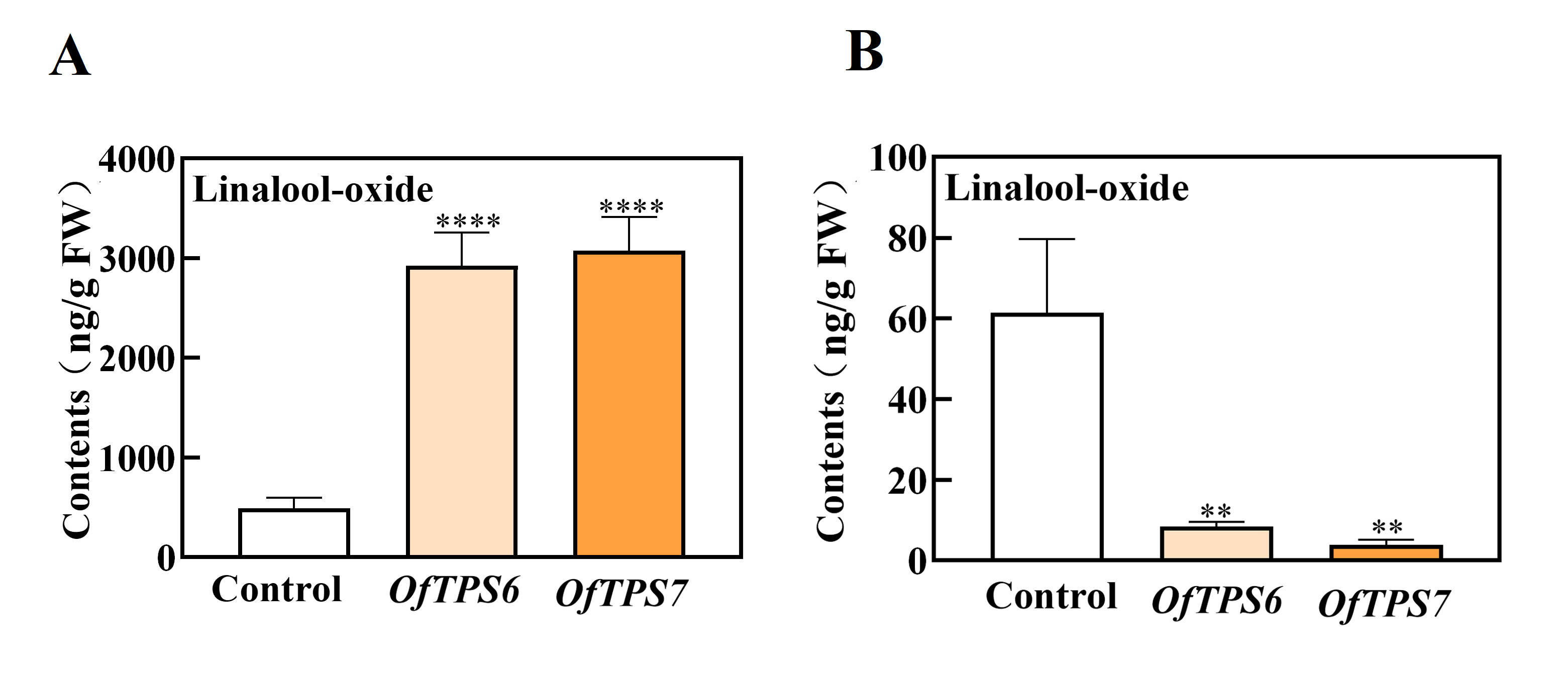

Supplement: Web_Material_uhaf155 [file web_material_uhaf155.zip › Suplemental Figure 9.jpg]

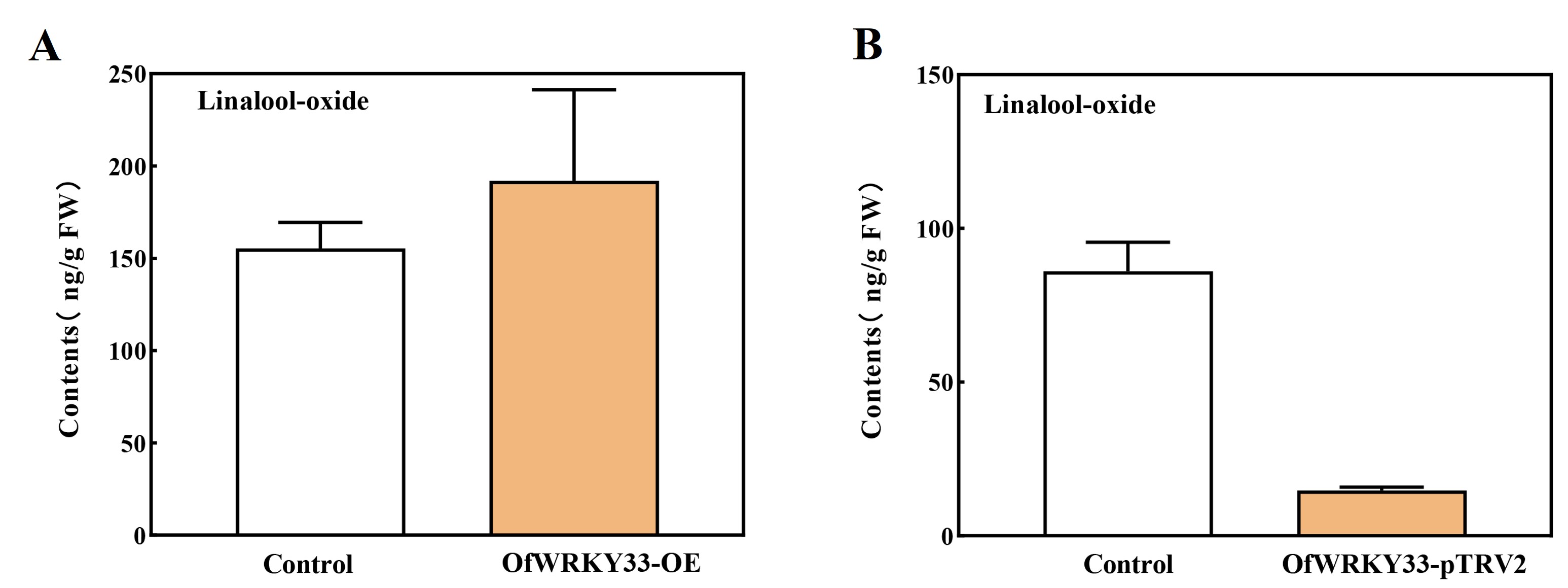

Supplement: Web_Material_uhaf155 [file web_material_uhaf155.zip › Supplemental Figure11.jpg]

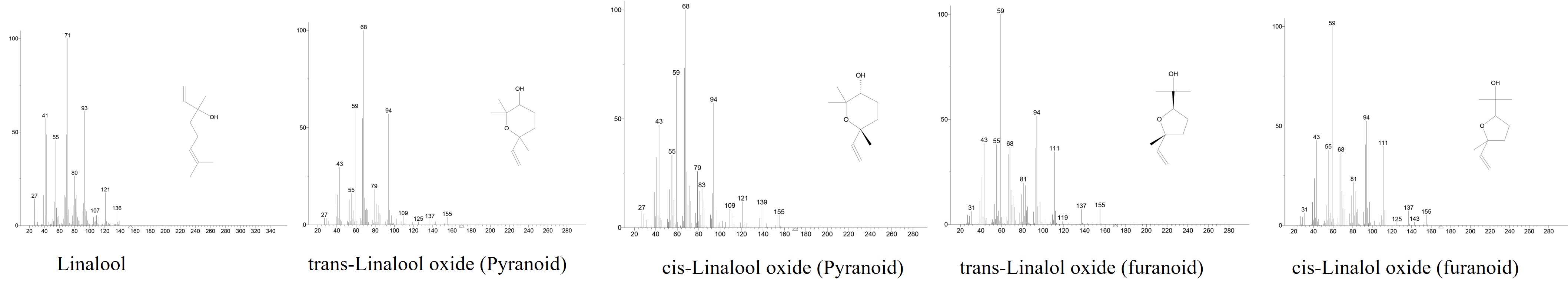

Supplement: Web_Material_uhaf155 [file web_material_uhaf155.zip › Supplemental Figure 1.jpg]

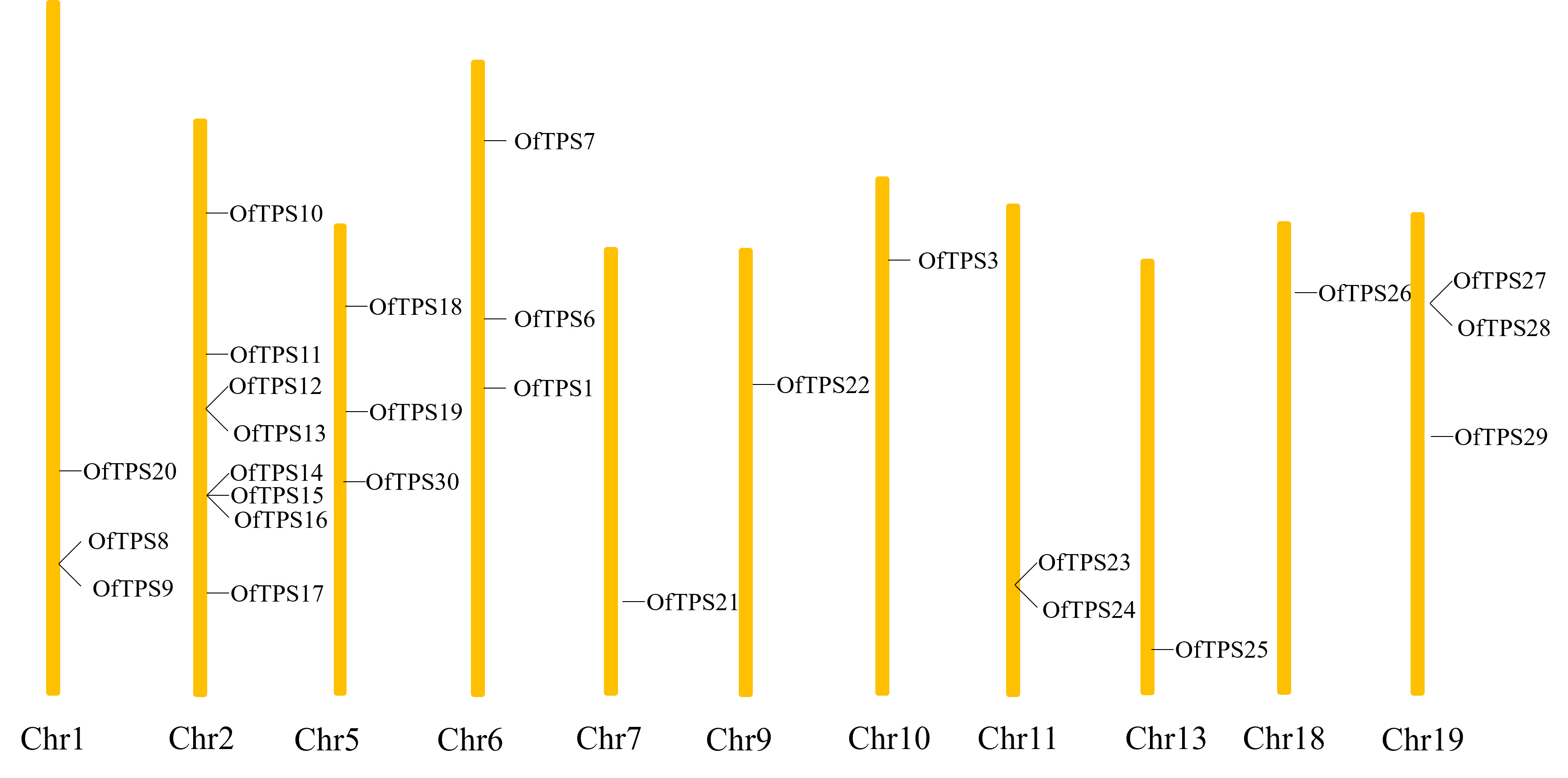

Supplement: Web_Material_uhaf155 [file web_material_uhaf155.zip › Supplemental Figure 2.jpg]

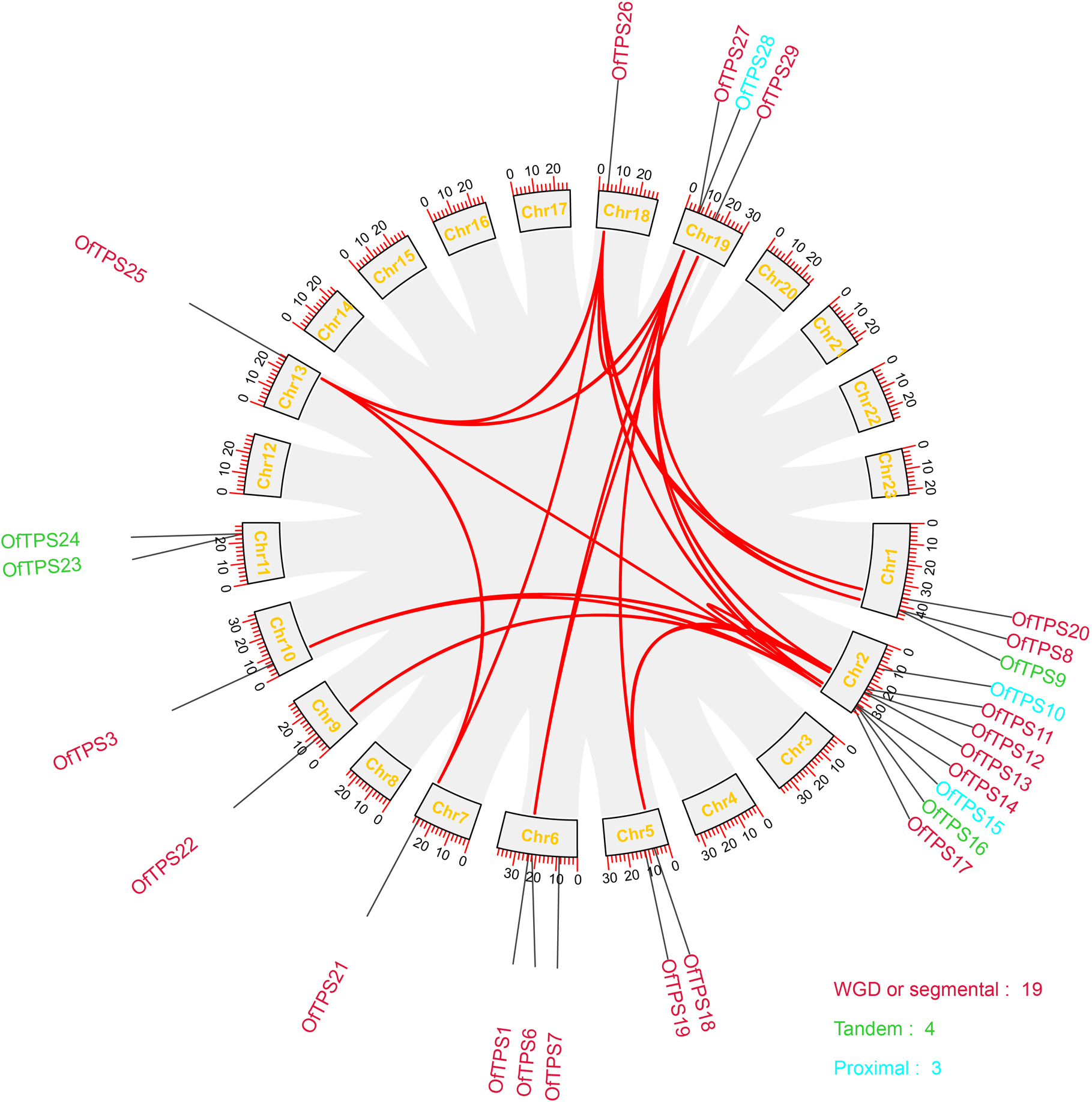

Supplement: Web_Material_uhaf155 [file web_material_uhaf155.zip › Supplemental Figure 3.jpg]

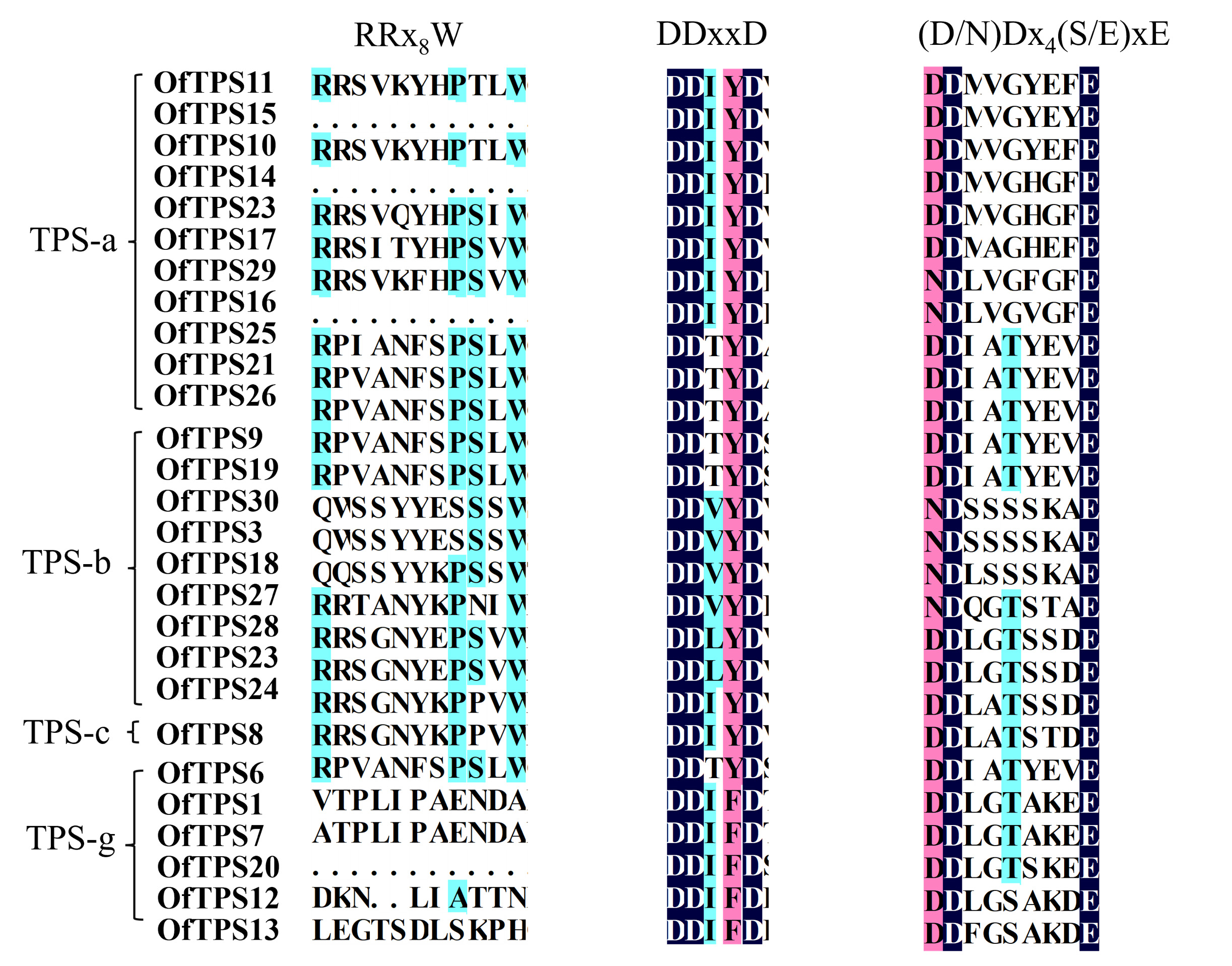

Supplement: Web_Material_uhaf155 [file web_material_uhaf155.zip › Supplemental Figure 4.jpg]

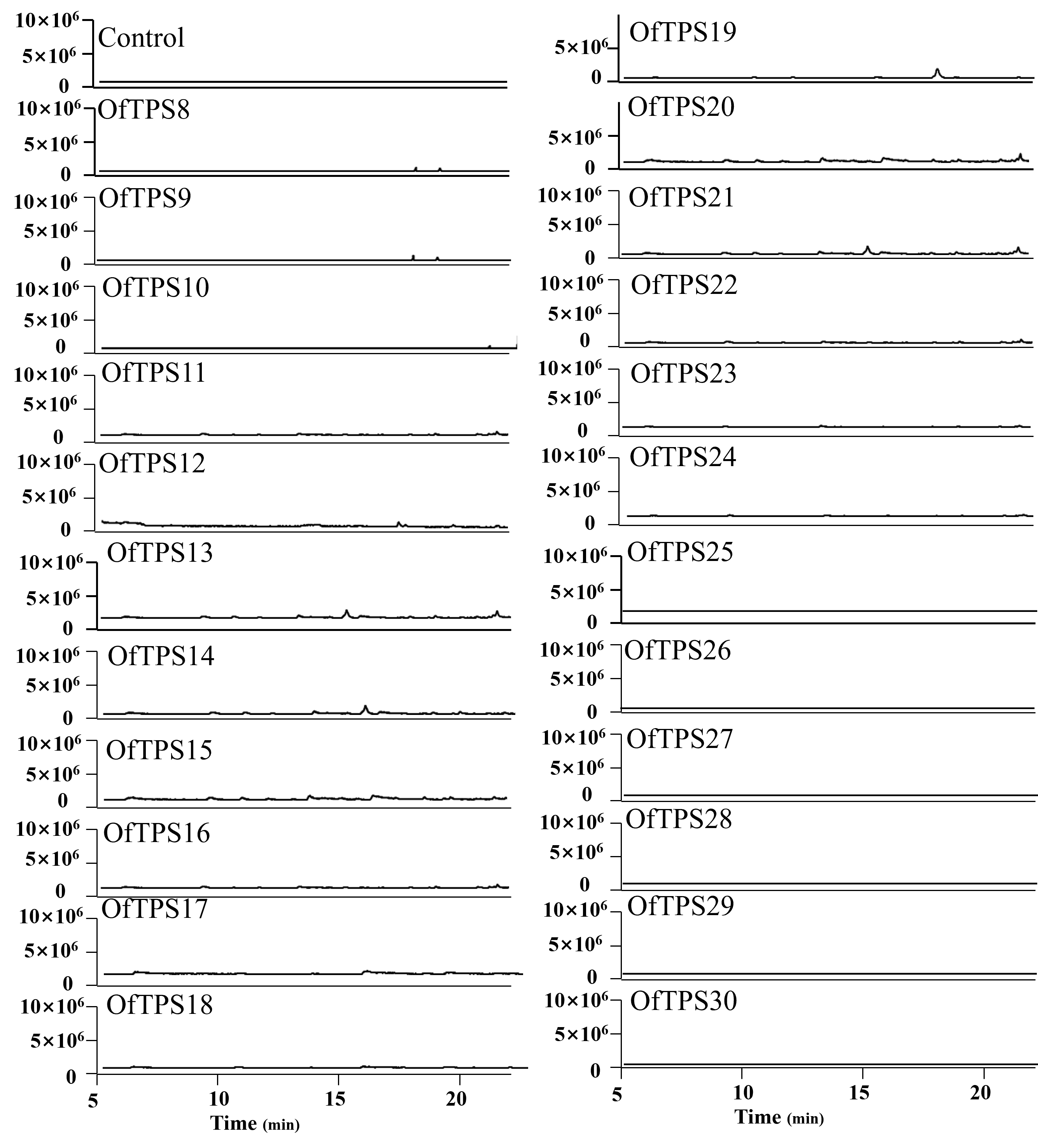

Supplement: Web_Material_uhaf155 [file web_material_uhaf155.zip › Supplemental Figure 5.jpg]

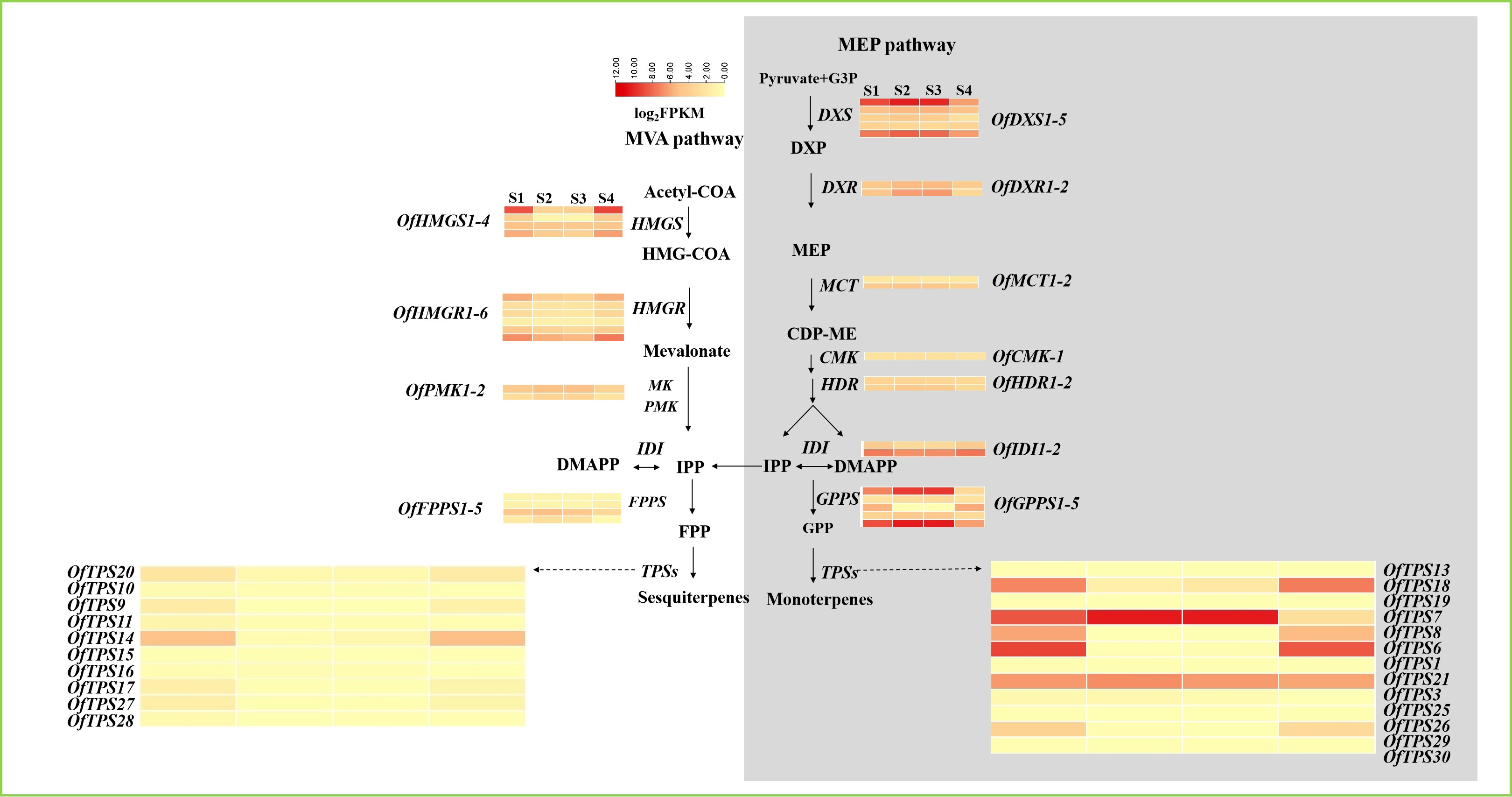

Supplement: Web_Material_uhaf155 [file web_material_uhaf155.zip › Supplemental Figure 6.tif]

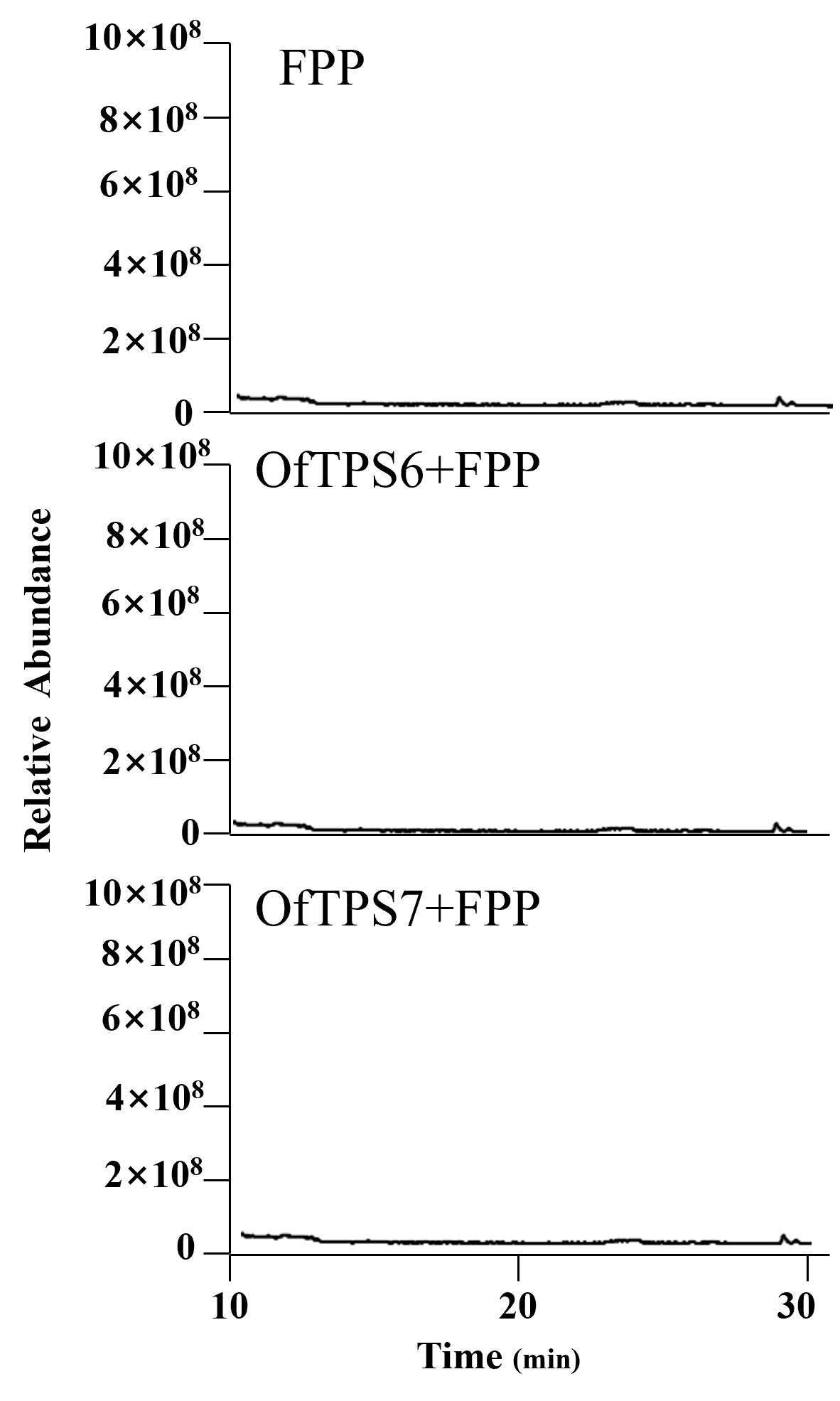

Supplement: Web_Material_uhaf155 [file web_material_uhaf155.zip › Supplemental Figure 7.jpg]

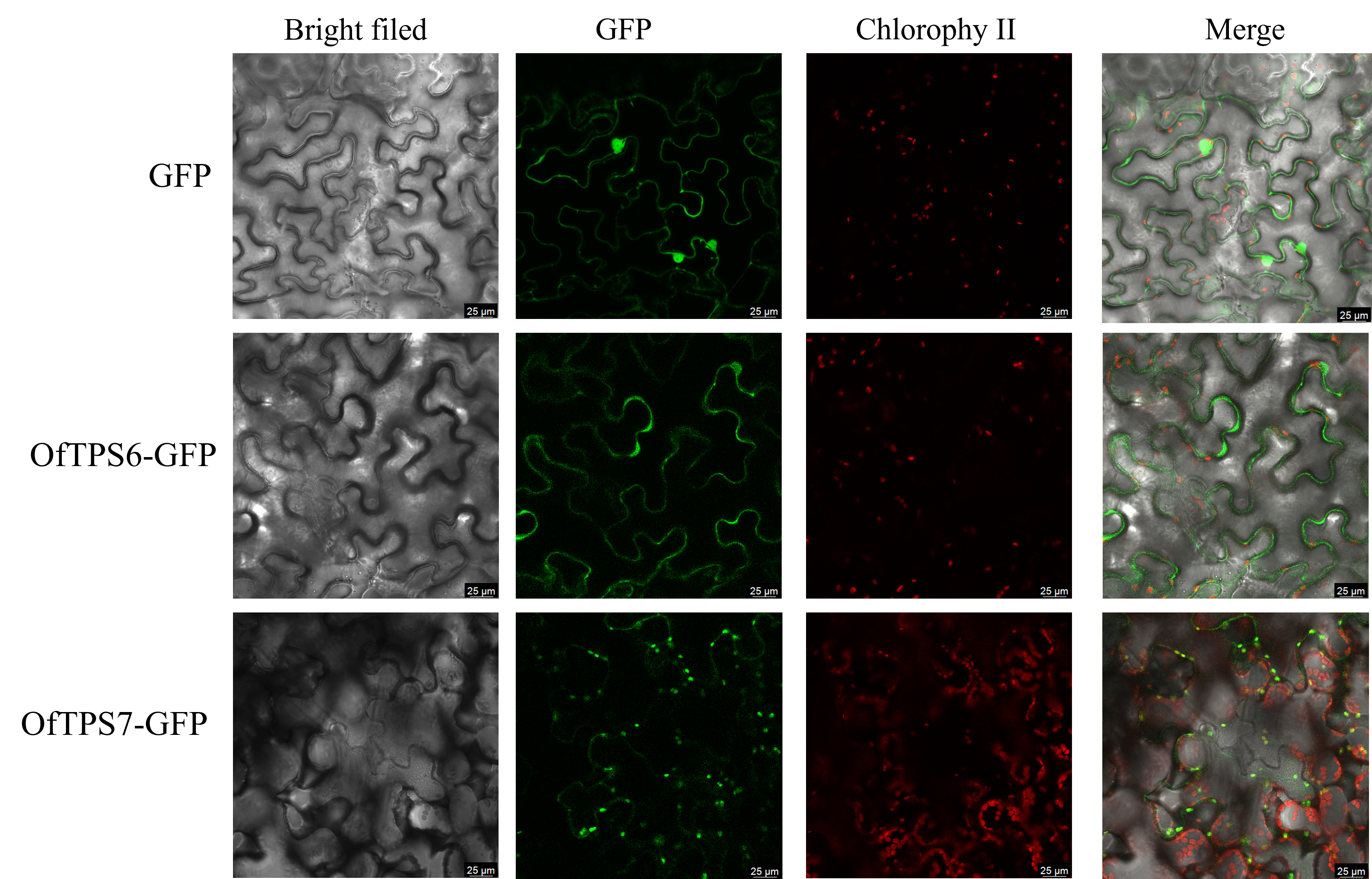

Supplement: Web_Material_uhaf155 [file web_material_uhaf155.zip › Supplemental Figure 8.jpg]

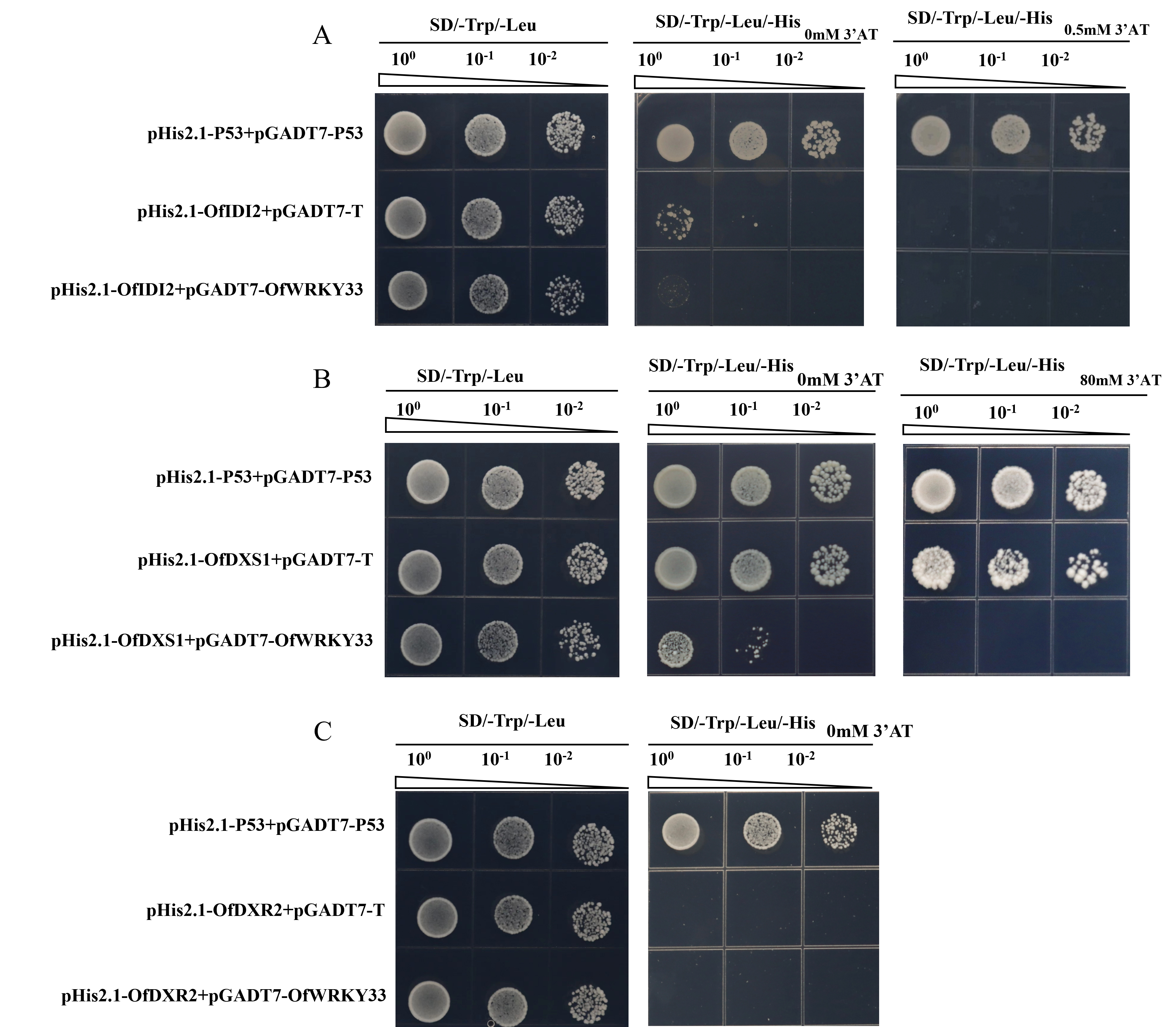

Supplement: Web_Material_uhaf155 [file web_material_uhaf155.zip › Supplemental Figure 12.jpg]

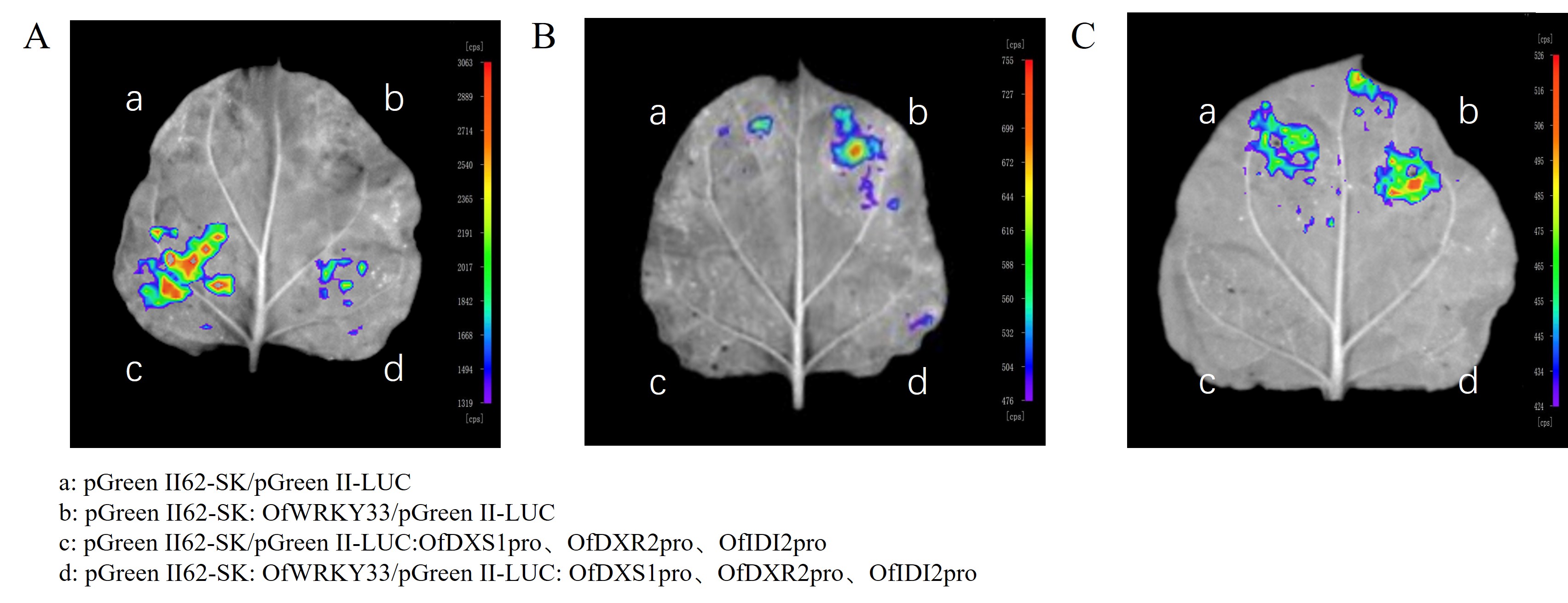

Supplement: Web_Material_uhaf155 [file web_material_uhaf155.zip › Supplemental Figure 13.jpg]

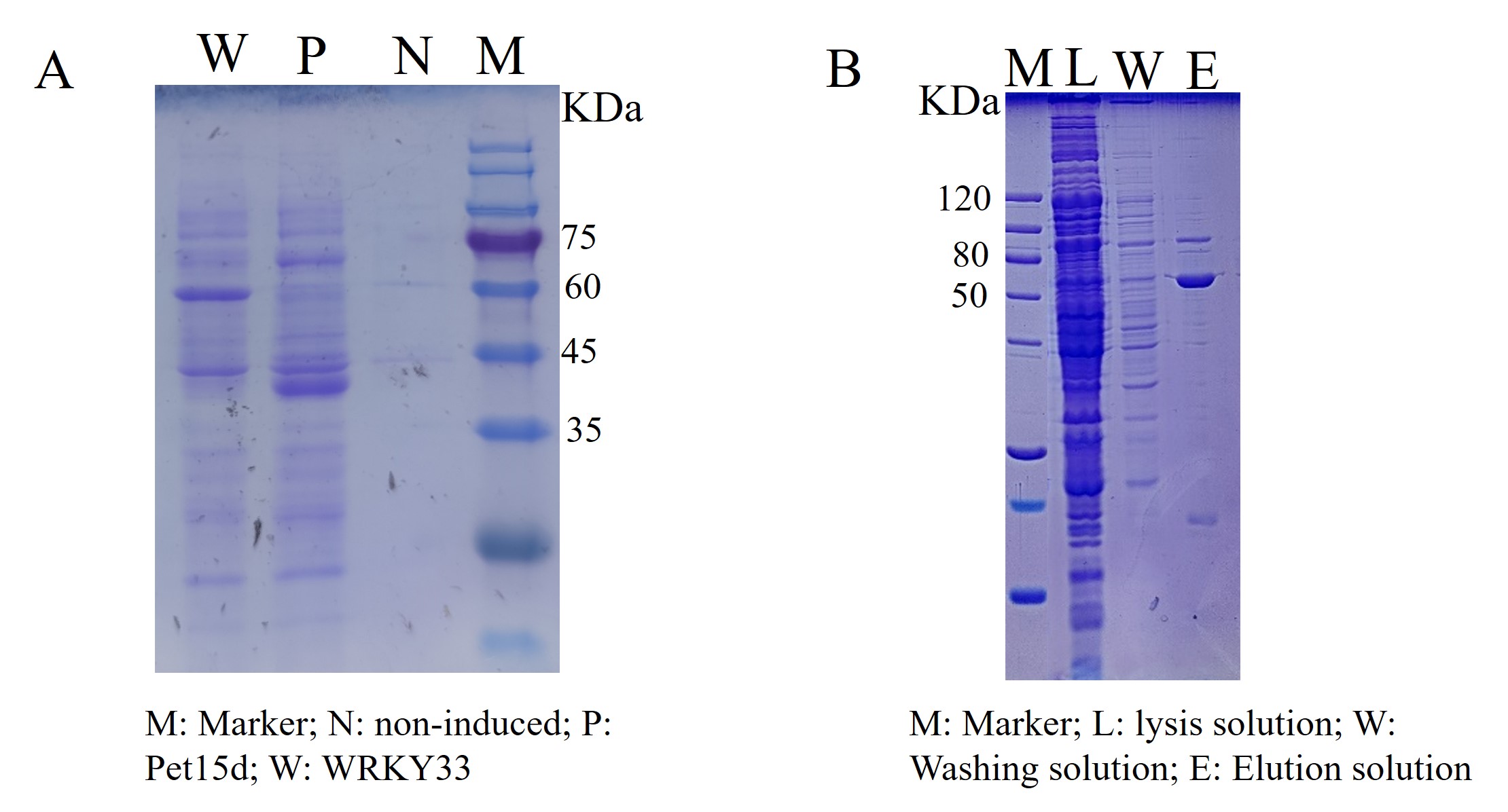

Supplement: Web_Material_uhaf155 [file web_material_uhaf155.zip › Supplemental Figure 14.jpg]

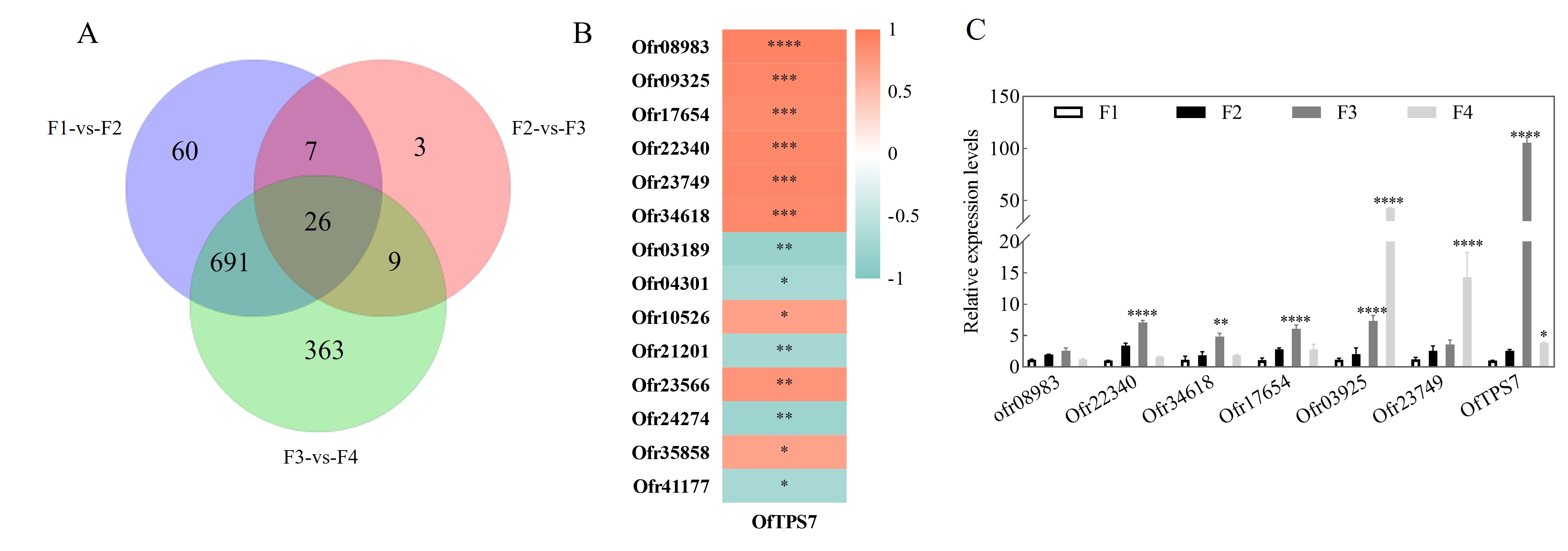

Supplement: Web_Material_uhaf155 [file web_material_uhaf155.zip › Supplemental Figure 10.jpg]
